# Anti–Methicillin-Resistant *Staphylococcus aureus* Nanoantibiotics

**DOI:** 10.3389/fphar.2019.01121

**Published:** 2019-10-04

**Authors:** Raphaël Labruère, A. J. Sona, Edward Turos

**Affiliations:** ^1^Institut de Chimie Moléculaire et des Matériaux d’Orsay (ICMMO), CNRS, Univ Paris Sud, Université Paris-Saclay, Orsay, France; ^2^Center for Molecular Diversity in Drug Design, Discovery and Delivery, Department of Chemistry, University of South Florida, Tampa, FL, United States

**Keywords:** nanoparticle antibiotics, nanoparticles, nanoantibiotics, methicillin-resistant *Staphylococcus aureus*, MRSA

## Abstract

Nanoparticle-based antibiotic constructs have become a popular area of investigation in the biomedical sciences. Much of this work has pertained to human diseases, largely in the cancer therapy arena. However, considerable research has also been devoted to the nanochemistry for controlling infectious diseases. Among these are ones due to bacterial infections, which can cause serious illnesses leading to death. The onset of multi-drug-resistant (MDR) infections such as those caused by the human pathogen *Staphylococcus aureus* has created a dearth of problems such as surgical complications, persistent infections, and lack of available treatments. In this article, we set out to review the primary literature on the design and development of new nanoparticle materials for the potential treatment of *S. aureus* infections, and areas that could be further expanded upon to make nanoparticle antibiotics a mainstay in clinical settings.

## Introduction

The continuing exploration and expansion of new nanotechnologies have opened up a broad range of potential applications and areas of investigation within the biomedical sciences. Two of the more prominent arenas for exploration are in drug delivery and new therapeutics. Of most interest to us is the application of nanoscience and nanotechnology for control of infectious diseases. Among the most aggressive, yet common, human pathogenic agents is a bacterium called *Staphylococcus aureus* (abbreviated as *S. aureus* or staph) (Tong et al., 2015). A ubiquitous Gram-positive microbe, *S. aureus* has adapted to a wide variety of species, including the human host, where it comprises the natural flora on the surface of human skin. The passage of *S. aureus* between individual organisms occurs upon contact, either through skin-to-skin or by way of contaminated object-to-skin transmission, as well as (to a lesser degree) aerobically, *via* coughing or sneezing. Staphylococcal skin infections are common and cause both minor incidents, such as skin blemishes and pimples, as well as more painful and concerning issues, such as skin boils, impetigo, abscesses, and cellulitis folliculitis. In most cases, staph infections can be treated effectively with commercial antibiotics applied dermally or orally. Although most individuals are healthy carriers largely unaffected by the innocuous presence of *S. aureus*, invasion of the microbe through the epithelial or mucosal surface through cuts or open sores in the skin can enable the bacteria to get into the bloodstream (McCaig et al., 2006) and lead to serious illnesses, which range from skin and soft tissue infections to debilitating and very often deadly infections in the blood, bone, brain, and vital internal organs (Lowy, 1998; Crossley et al., 2009). It is therefore imperative that even minor staphylococcal infections be treated aggressively before they spread to other tissues and lead to sepsis upon release of toxins. *S. aureus*–necrotizing pneumonia produces bacterial abscesses in the lung, *S. aureus* endocarditis may cause death through heart failure, and *S. aureus* meningitis has high rates of mortality, while osteomyelitis is the result of *S. aureus* infiltrating into bone (Sibbald et al., 2006). The deadly nature of *S. aureus* is attributable to the release of bacterial toxins, including α-, β-, γ-, and δ-toxins, exfoliatin, enterotoxins that cause toxic shock and scalded skin syndrome, and poisoning from infected food (Dinges et al., 2000). Hospital-acquired, or nosocomial, infections are common to virtually every emergency, surgical, and recovery room; patient care centers and long-term health care facilities. Nosocomial infections from *S. aureus* arise from surgery, implantations, catheters or skin ports, open wounds, as well as drug- or disease-induced immunosuppression (McCaig et al., 2006).

Antibiotic resistance in disease-causing bacteria such as *S. aureus* has become a major medical issue not only because of their rise in incidence but also in their increasing degree of antibiotic resistance and lethality (Klevens et al., 2007). Antibiotic-resistant *S. aureus* is the result of widespread, often poorly controlled, use of antibacterial antibiotics since the 1940s (Mcdougal et al., 2006). Initially, the gravest concern was in hospitals, where large numbers of sick people, surgical interventions, and antibacterial agents converge. However, there are increasing issues over drug-resistant pathogens entering the open community beyond health care centers and emergency rooms (Walsh, 2004). Drug-resistant infections claim tens of thousands of lives annually in the United States, and antibiotic therapy has been reduced to only a few approved therapies (Walsh, 2003; Norrby et al., 2005).

**Figure 1:**
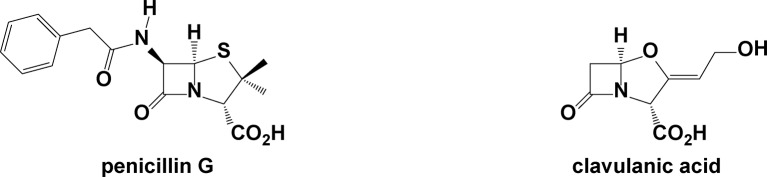
Structures of two commercial β-lactams, penicillin G and clavulanic acid.

Penicillin, the formative member of the β-lactam family of antibacterial drugs used extensively for decades to treat a wide assortment of bacterial infections and diseases, is largely the culprit for this worldwide epidemic. The acquisition and transmission of antibiotic-resistance genes is the microbe’s evolutionary response to the lethal effects of β-lactam drugs. The ability to kill *S. aureus* quickly with penicillin led to the development of a large number of other β-lactam compounds with increasing strength and spectrum of antibiotic activity. Among these was a penicillin analogue known as methicillin. Almost immediately upon introduction into the clinic, physicians began noticing strains of *S. aureus* in patients demonstrating elevated resistance to methicillin, bringing awareness of a dangerous new form of the microbe called methicillin-resistant *Staphylococcus aureus*, or MRSA (Barber, 1961; Jevons, 1961). An underlying cause of methicillin resistance in *S. aureus* is *mecA*, an acquired gene responsible for the production of penicillin binding protein 2a. This protein is a structurally modified form of the transpeptidase that creates the peptidyl cross-links in the bacterial cell wall but offers lower binding affinity for methicillin and other similar β-lactam antibiotics (Chambers, 1997). Many MRSA strains also are able to express high levels of hydrolytically efficient penicillinases (or β-lactamases), proteins capable of hydrolyzing β-lactam drugs to inactive ring-opened products. β-Lactamase–producing MRSAs are less sensitive to β-lactam antibiotics in the absence of β-lactamase inhibitors, such as clavulanic acid (**Figure 1**).

**Figure 2:**
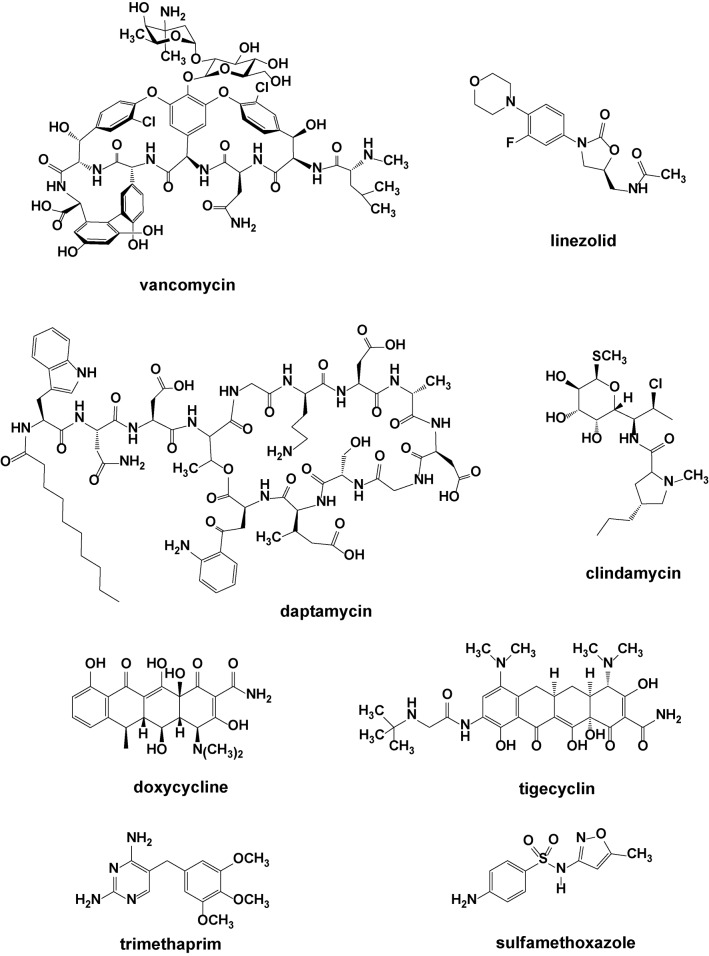
Structures of various commercial antibacterial agents.

In addition to the ability of *S. aureus* to rapidly acquire genes for drug resistance, many antibiotics are also inherently highly water-soluble and thus not very effective for treating infections in fatty tissue, in biofilms, or along the surfaces of bone or implanted devices, where drug-resistant bacterial infections frequently occur (Costerton and Stewart, 1999). The most effective antibiotics for MRSA eradication are vancomycin, linezolid, and a few others in combination with vancomycin (**Figure 2**). Daptomycin, clindamycin, doxycycline, tigecyclin, and trimethoprim–sulfamethoxazole combo are also efficient against most MRSA strains (Deresinski, 2005). Like the β-lactam antibiotics, vancomycin targets bacterial cell wall (murein) biosynthesis. However, where the β-lactams block the cross-linking transpeptidase proteins themselves (through covalent modification of an active site serine), vancomycin and daptomycin instead act on the murein itself, not the enzymes responsible for its construction. Specifically, these two macrocyclic compounds wrap themselves around the D-alanyl-D-alanine terminal residues on the glycopeptide, through tight hydrogen bonding, and effectively obscure that unit from the transpeptidase enzyme (Williams, 1996). About 15 years ago, the first vancomycin-resistant *S. aureus* (VRSA) isolates were identified in the United States (Chang et al., 2003).

One of the frustrations and concerns many physicians and patients have about staphylococcal infections, other than those mentioned earlier, is that they are often recurring and cause frequent relapses in patients. This arises largely from *S. aureus*’s ability to infect various types of human cells, where it can lie in a dormant state for months to even years (Lowy, 2000; Ellington et al., 2003; Hess et al., 2003). While in dormancy, the bacteria are largely unresponsive to antibiotics. Effective treatment of recurring MRSA infections, similar to those of viral infections, is therefore very challenging and may in fact not be truly achievable (Ellington et al., 2006; Dancer, 2008). *S. aureus* outfits itself with a selection of efficient virulence factors, of which one is comprised of the surface adhesive molecules known as adhesins. Consequently, *S. aureus* cells are able to attach to biomolecules surrounding the matrix of host cells and to endocytosis receptors like β1-integrins on the surface of the host cells, enabling *S. aureus* to enter the host cell through clathrin-coated pits (Jevon et al., 1999). Staphylococcal intrusion can induce cytosis of the host cell by triggering cellular apoptosis or, conversely, deregulating the essential anti-apoptotic factors to keep the host cell alive but in a state of dormancy (Tuchscherr et al., 2011). *S. aureus* is able to avoid phagocytic engulfment into macrophages and thus survive in the human body without being immediately detected by the immune system (Goodyear and Silverman, 2003; Potempa and Pike, 2009).

All of these traits and clinical problems associated with *S. aureus* in general, and MRSA in particular, highlight the persistent need for new approaches for effectively dealing with MRSA infections. The implementation of nanoparticles as anti-infective materials and in antibiotic drug delivery may offer value in this regard (Huh and Kwon, 2011). The unique size and properties of nanoparticles may afford opportunities to enhance the effectiveness of antibacterial drugs whose clinical potential may otherwise suffer because of inadequate water solubility, pharmacokinetics, bioavailability, biostability, susceptibility to existing resistance mechanisms, or cellular or systemic toxicity (Webster, 2006).

A variety of scenarios already exist as to the usefulness of nanoparticles for antibacterial drug delivery or as bactericides, particularly with respect to MRSA. This literature review aims to outline the most salient efforts reported thus far toward these objectives.

## Types of Nanoparticles for Antibacterial Applications

The continuing interest in and development of nanoparticles for drug delivery applications have largely been focused in the area of cancer therapies. However, within this general domain is an emerging subset of work directed at the anti-infectives area, motivated by the above-described growing concerns over drug-resistant microbial infections and the recognition that antibiotics have inherent limitations. Collectively, the central premise in these investigations pertains to the design of new nanoparticle-based antibiotics, termed *nanoantibiotics*. For the purpose of this review, we arbitrarily restrict the use of the term *nanoantibiotics* to antimicrobial *particles measuring 10–350 nm in size*. Although particles larger or smaller than these specific dimensions may also be relevant to the discussion, it is this more narrow size range that defines the major work in anti-infective nanomedicines. In certain cases, we will mention research on larger particles if it seems relevant to do so.

**Figure 3:**
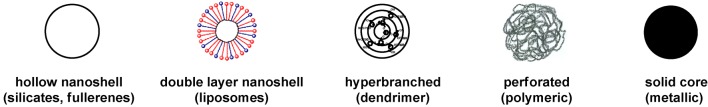
General types of nanoparticles used for antimicrobial applications.

The nanoparticle antibiotic may be an inert carrier of an antibiotic agent or possess antibiotic activity of its own. Most commonly, antibacterial properties arise from an attached (or entrapped) antibiotic substance that is bound to the nanoparticle’s surface or encapsulated inside and is transferred to the microbe through direct interaction. Our laboratory has previously reviewed the various types of nanoparticle constructs and approaches that have been investigated for anti-infective applications (Abeylath et al., 2009). Among these include the five generalized nanoparticle prototypes shown in **Figure 3**. Starting from the left, the simplest model for drug-carrying vehicles would be the hollow nanoshell, constructed of a polymeric material such as a silicate or carbon network that enables entrapment of the antibiotic substance within the empty cavity. Closely related are the double layer nanoshell vesicles comprised of self-assembled lipid bilayers, forming a liposome. Not too distant from these are the hyperbranched and perforated nanoparticles based on dendritic or polymeric motifs that enable small molecules to be held within the crevices of the matrix, either through non-covalent or covalent means. On the far end of the spectrum are the completely solid core nanoparticles, made of metals such as silver or gold, which cannot carry a payload internally but can be adorned on the surface with antimicrobial ligands.

**Figure 4:**
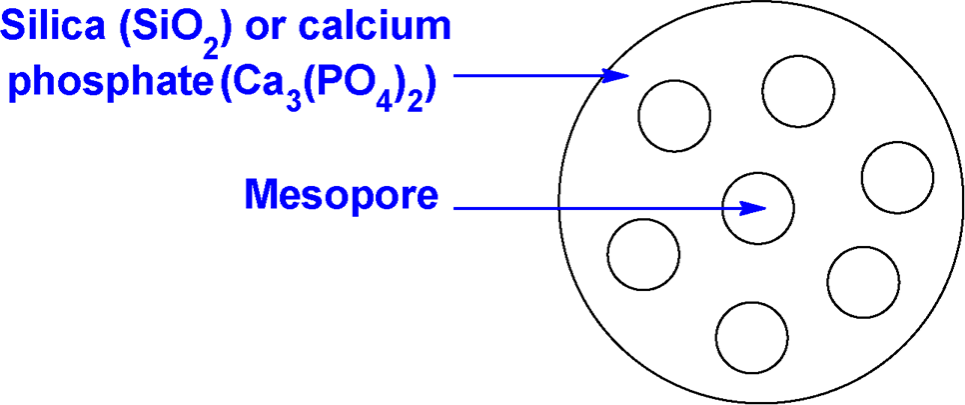
Schematic representation of a perforated (porous) inorganic nanoparticle.

We should next talk about the materials commonly used to form the nanoparticle matrix, as they may be an inert metal such as gold or silver, or a synthetic inorganic or organic substance, or even one that is biologically produced. As will be presented, a broad assortment of inorganic and organic molecules or materials have already been used to construct these different types of nanoparticles, and their intended biomedical applications have ranged from delivery of pharmaceutical drugs and genes to diagnostic imaging agents, photothermal therapy, and antiseptics (Sekhon and Kamboj, 2010a; Sekhon and Kamboj, 2010b). For instance, hollow (non-porous) nanoshells and perforated (porous) nanoparticles have been constructed from silica and calcium phosphate (**Figure 4**) (Son et al., 2007a; Son et al., 2007b; Blanco et al., 2015; Tang et al., 2015; Wang et al., 2015).

**Figure 5:**
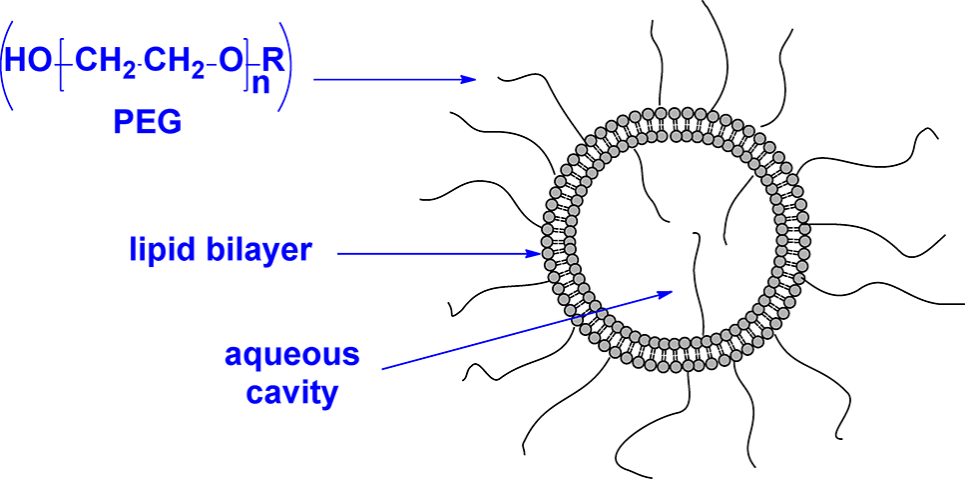
Schematic representation of a polyethylene glycol (PEG)–coated liposome.

Liposomes have been studied as nanoparticle-sized carriers of biologically active compounds for over four decades and evaluated as such for drug delivery applications (Pinto-Alphandary et al., 2000; Tang et al., 2015). Liposomes are constructed from phospholipids that readily form bilayers separating an aqueous phase on the inside from the bulk aqueous media on the outside, with a water-soluble drug contained within the aqueous core or a lipophilic drug sequestered in the lipophilic bilayer. Liposomes are the most widely used nanoparticle vehicle for antimicrobial drug delivery (Pinto-Alphandary et al., 2000; Matthews et al., 2010; Zhang et al., 2010). It is generally accepted that liposomes interact with the cell membrane, through a mediated or passive fusion process that permits the release of the encapsulated agent directly into the membrane of the target cell (Daraee et al., 2016). One of the practical limitations in the use of liposomes is their osmotic fragility that can allow the entrapped species to leak through the bilayer (Pattni et al., 2015). The exterior surface of the liposome can be further functionalized with polyethylene glycol (PEG) to provide stealth capabilities in the human body, avoiding clearance by the reticuloendothelial system (RES) and increasing blood circulation times (**Figure 5**) (Mei et al., 2014). The PEG surface coating also creates an ordered hydration layer around the particle, which inhibits undesired binding with plasma proteins.

**Figure 6:**
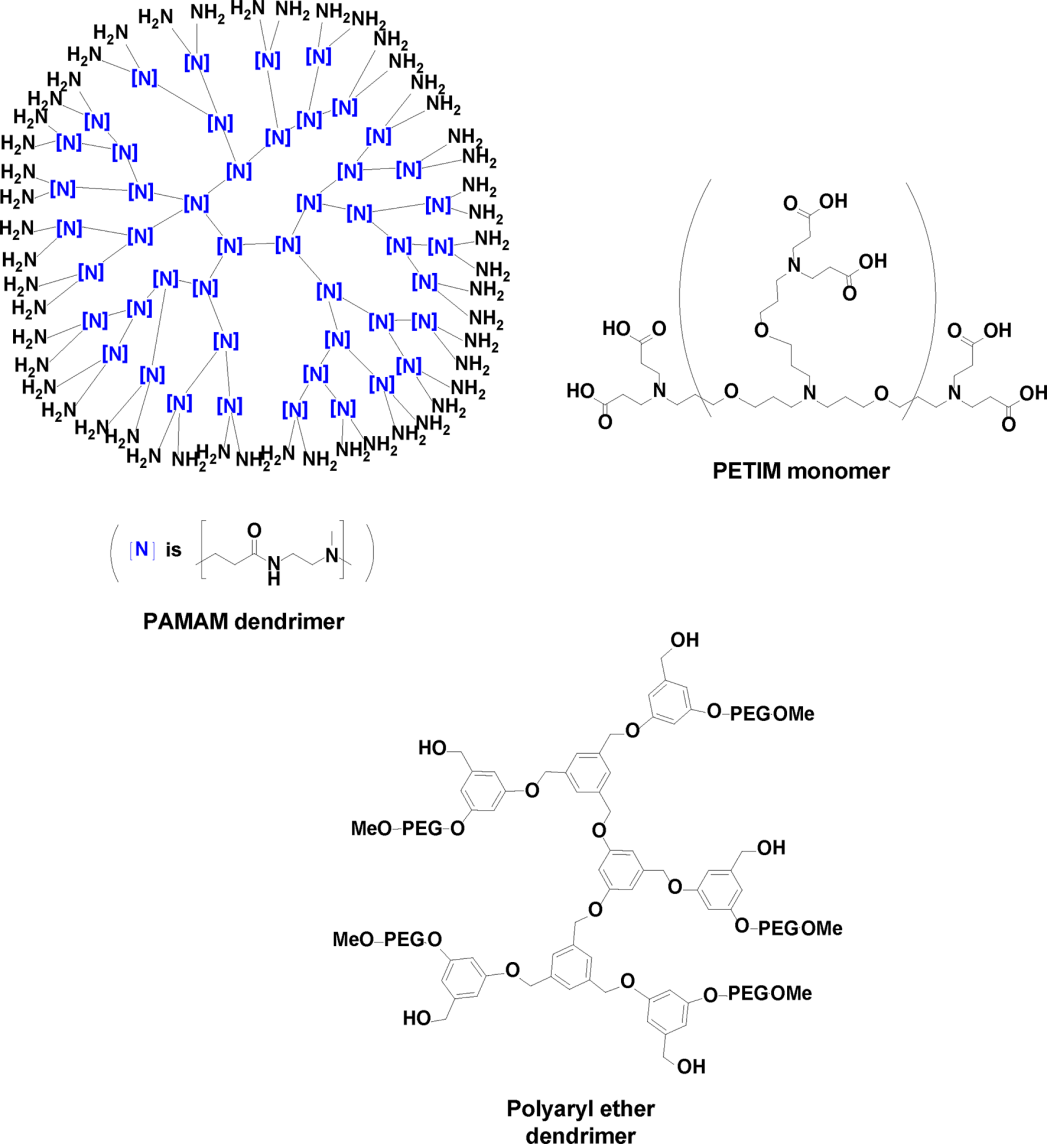
Three dendrimeric constructs used for drug delivery.

Dendritic polymers, or *dendrimers*, have likewise been extensively explored over the last several decades for drug delivery purposes (Gillies and Fréchet, 2005; Kesharwani et al., 2017). These hyperbranched molecules create interior crevices amenable to encapsulation of guest molecules. Through chemical synthesis, heteroatomic functionality (such as amines, carbonyls) can be incorporated as a handle for better sequestering specific drug molecules through entrapment or covalent attachment (**Figure 6**) (Buhleier and Vogtle, 1978). Balogh reported polyamidoamine (PAMAM) dendrimer–silver complexes that have strong antimicrobial activity against various Gram-positive bacteria through a slow release of silver ions (Balogh et al., 2001). Poly(propyl ether imine) (PETIM) silver complexes have a better bioavailability compared with PAMAM and were found to be non-cytotoxic even at concentrations that would exhibit activity against *S. aureus* and MRSA (Jain et al., 2010; Suleman et al., 2015). Dendrimers containing quaternary ammonium salts as functional end groups exert antimicrobial behavior by disrupting bacterial membranes (Xue et al., 2015). The unusually high local concentration of the polycationic structure of biocidal dendrimers induces a strong electrostatic adsorption onto the negatively charged bacterial lipid bilayer, increasing membrane permeability and leakage of potassium ions through the bacterial cell membrane (Cheng et al., 2008).

**Figure 7:**
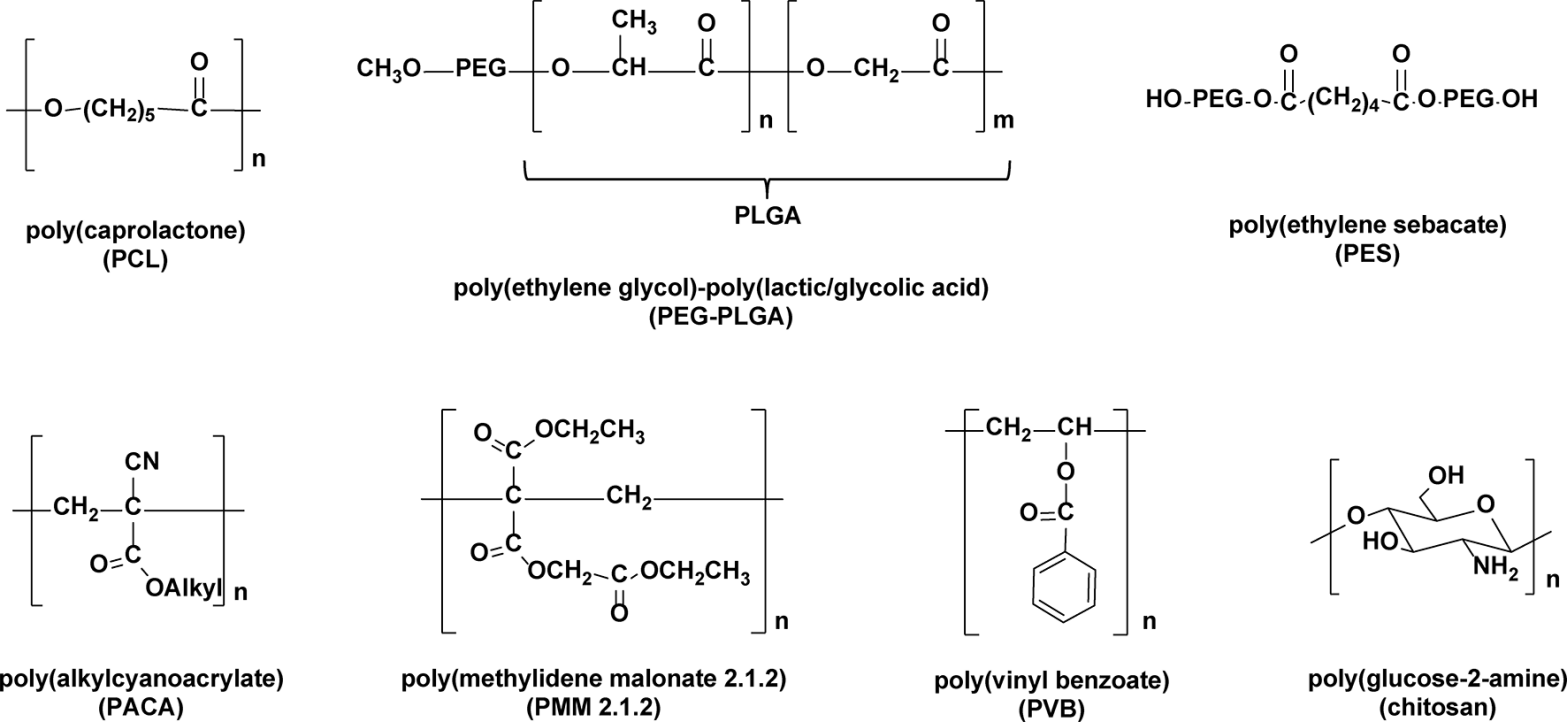
Polymeric frameworks commonly used as nanoparticle matrices.

Polymer-based nanoparticles constitute another major type of delivery vehicle for a wide assortment of drugs. Biocompatible and biodegradable polymers have been used extensively in the clinic for controlled drug release (Soppimath et al., 2001; Kumari et al., 2010; Mora-Huertas et al., 2010; Liao et al., 2014; Ulbrich et al., 2016). The most commonly used biodegradable polymers for nanoparticle drug delivery are poly(lactic acid) (PLA), co-polymers of lactic/glycolic acids (PLGAs) and poly(alkyl cyanoacrylates) (PACA), poly(caprolactone) (PCL), chitosan, and gelatin (**Figure 7**) (Kohane and Langer, 2010). In addition, the biodegradability of some new nanopolymers has been clearly established (Visage et al., 2001; Guhagarkar et al., 2009; Labruère et al., 2010). Biocompatible and biostable polymers have been also used in nanoparticle formulations (Uhrich et al., 1999; Kohane and Langer, 2010).

Polymeric nanoparticles possess several unique characteristics, including structural stability and a narrow size distribution. Their properties such as size, zeta potentials, and drug release profiles can be precisely tuned by varying the lengths of the polymer, the choice of surfactant, and the selection of the organic solvents during the synthesis. Apart from this, these nanoparticles are typically stable in blood and, by pegylation on the surface, can delay elimination by the RES to deliver small molecule drugs, proteins, peptides, or nucleic acids (Abeylath et al., 2009; Huh and Kwon, 2011). Many of the biodegradable polymeric nanoparticles are capable of drug release from the nanoparticle through hydrolysis (Kamaly et al., 2016).

Gold nanoparticles have generated considerable interest within the biomedical community for *in vivo* imaging, drug delivery, and thermotherapy for the localized killing of cancer cells (Pissuwan et al., 2010; Vigderman and Zubarev, 2013; Li et al., 2014), while silver nanoparticles are of value for their innate antimicrobial properties (Rai et al., 2009; Samberg et al., 2011; Durán et al., 2016). Hybrid nanoparticles comprised of inorganic materials with organic pharmaceuticals have shown utility for targeted imaging and therapy, drug and gene delivery, and regenerative medicine (Xu et al., 2006; Bandow and Metzler-Nolte, 2009).

## Nanoparticles as Delivery Vehicles of Anti-MRSA Agents

For several decades, attempts have been made to enhance the bioactivity and effectiveness of various pharmaceutical agents through the use of nanoparticle delivery methods. The concept is a straightforward one, to take advantage of the nanoparticle size, shape, charge, or protective inner environment as a means to better introduce the agent into a cell, organ, or tissue, or through a delayed release, such that the compound can more effectively exert its biological effect. The range of possibilities for achieving this is extensive, for the agent can be introduced through entrapment or encapsulation, adsorption, or chemical attachment within the nanoparticle matrix. It is the intention, of course, that depending on the nanoparticle material, its size or charge, its stability, and its sensitivity toward chemical or biological degradation, the pharmacokinetics and therapeutic index of the pharmaceutical agent can be significantly improved. In general, nanoparticles may improve serum solubility, prolong the systemic circulation lifetime, and release drugs at a sustained and controlled manner, better than alternative means such as formulation or capsule-based delivery. Additionally, nanoparticles can usually enter host cells through endocytosis and thus be used to treat intracellular microbial infections. Several nanoparticle-based drug delivery systems are under investigation, or have already been approved, for clinical use (Faraji and Wipf, 2009).

The first report of an antibiotic in a nanoparticle for use against MRSA appeared in 1994 (Onyeji et al., 1994). Multilamellar liposomes containing either vancomycin or teicoplanin were found to have enhanced *in vitro* activity against MRSA located within human macrophages. The viability of MRSA inside of macrophages has more recently been evaluated using vancomycin-encapsulated liposomes having an average diameter of 250 nm. Whereas vancomycin alone was unable to kill MRSA-infected macrophages, presumably due to poor access, treatment with liposomes containing vancomycin (200 and 800 μg/ml concentrations) resulted in a marked reduction in MRSA survival. However, by comparison, treatment of MRSA-infected macrophages with surface-pegylated liposomes containing the same amount of vancomycin failed to alter MRSA viability. The inability of the pegylated liposomes to exert intracellular anti-MRSA activity is likely the result of the “stealth” effect by nanoparticle pegylation toward macrophages (Pumerantz et al., 2011). In a more recent investigation, a different liposomal vancomycin formulation was found to be more effective *in vitro* against MRSA, and also in a murine MRSA infection model, compared to a similar dose of the non-liposomal vancomycin control (Sande et al., 2012).

Hollow inorganic nanoparticles comprised of hydroxyapatite have also been examined to help deliver vancomycin to the bone, and to also serve as a bone substitute material, for the *in vitro* and *in vivo* treatment of MRSA-induced chronic osteomyelitis (available from Berkeley Advanced Biomaterials with an average particle size of 100 nm). Vancomycin-loaded hydroxyapatite pellets are reported to release high levels of the antibiotic over a prolonged period, to provide effective local antimicrobial activity, and to stimulate the reconstruction of new bone in rabbit models of chronic osteomyelitis (Jiang et al., 2012). In a separate study, vancomycin entrapped in poly(終-caprolactone) microparticles (average diameter > 1,000 nm) reduced the required dosage of vancomycin to treat MRSA osteomyelitis (Ray et al., 2005; Cevher et al., 2006) and infective discitis (Wang et al., 2011).

**Figure 8:**
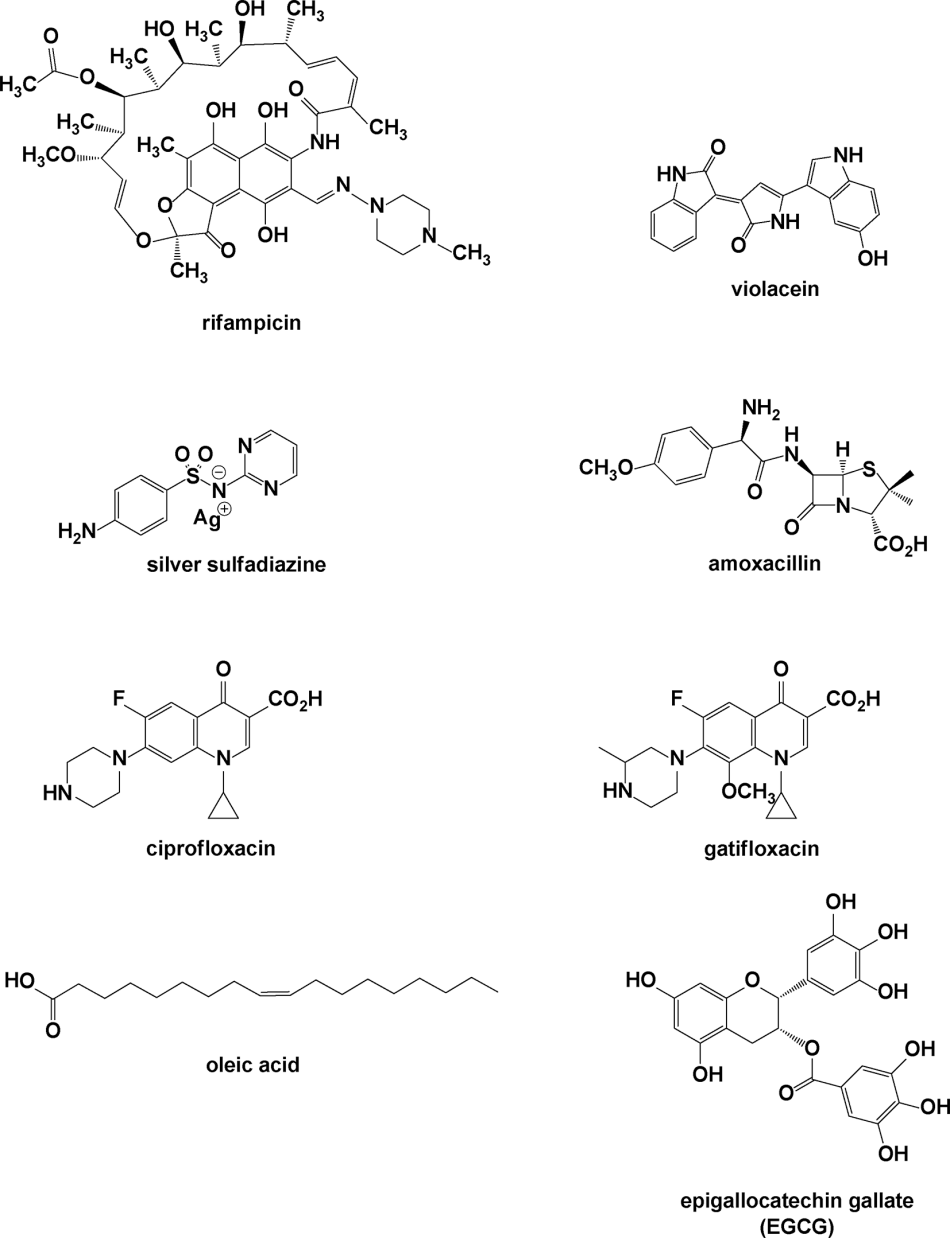
Molecules incorporated into nanoparticles.

Esmaeili et al. utilized PLGA nanoparticles to load a different antibiotic, rifampicin, by an emulsification/solvent diffusion method (Esmaeili et al., 2007). Rifampicin is typically used to treat *Mycobacterium* infections, including tuberculosis and leprosy, and may also be used with fusidic acid for MRSA infections (**Figure 8**).

**Figure 9:**
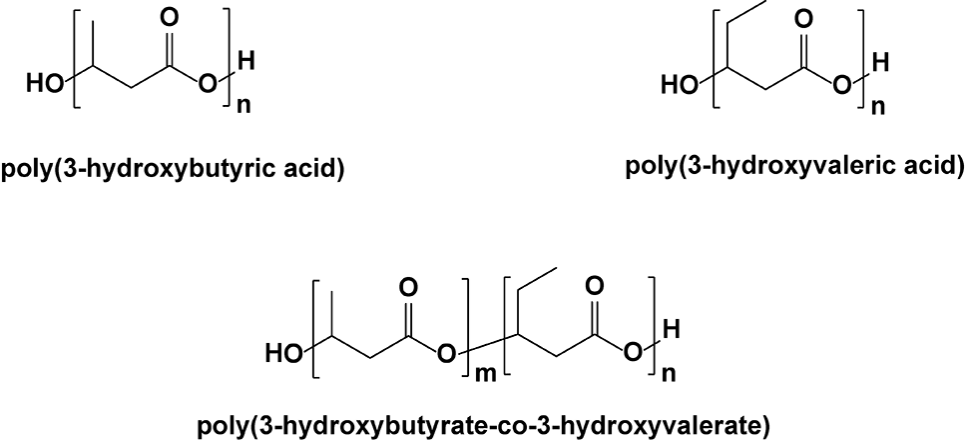
Three polyester frameworks used to construct microparticles for antibacterial delivery.

The rifampicin-loaded nanoparticles had an average size of 250 nm, with a relatively low capacity for drug loading within the inner crevices of the polymeric matrix. It appears that much of the initial burst release (>14% of the contained antibiotic) is due to diffusional release of rifampicin on or near the surface of the nanoparticles. *In vitro* antibacterial activity against a clinical strain of MRSA indicated that the minimum inhibitory concentration (MIC) decreased by a factor of four (0.002 μg/ml) compared to the free drug (0.008 μg/ml), suggesting that the nanoparticles may improve the delivery of the compound into the bacterial cells. In a similar study published in 2008, Durán et al. showed that rifampicin incorporated into poly(3-hydroxybutyrate-co-3-hydroxyvalerate) microparticles, ranging from 20 to 60 μm, maintained the anti-MRSA bioactivity, decreasing the cytotoxicity of rifampicin (**Figure 9**) (Durán et al., 2008).

Martins et al. have described the incorporation of violacein, an antibiotic isolated from *Chromobacterium violaceum*, into polymeric PLGA nanoparticles as a means to improve water solubility and possibly lower its cytotoxic effects for administration in biological systems (Martins et al., 2010). These were prepared by nanoprecipitation to afford a homogeneous size distribution of anionically charged nanoparticles of around 130 nm in diameter, with a high drug loading efficiency and sustained antibiotic release over 5 days. In assays against several MRSA strains, the violacein-entrapped nanoparticles exhibited at least a 50% reduction in the MIC values (1.2–2 µg/ml) compared to the free violacein (5.1 µg/ml) and were more effective at repressing bacterial growth over several days.

The use of silver compounds as a disinfecting agent is a time-tested staple of medicine. Silver nitrate (AgNO_3_) is still used clinically as an antibacterial agent, and compounds containing silver ion are often effective against both aerobic and anaerobic bacteria by precipitating bacterial proteins in the microbial respiratory chain. The combination of silver and sulfonamide provides silver sulfadiazine, which is used as a topical cream or aqueous colloid for treatment of burns. The emergence of antibiotic-resistant bacteria such as MRSA has led to a rejuvenation in the use of silver-based anti-infective materials (Madhumathi et al., 2010). Despite the popularity, there remains a level of scrutiny about whether silver-containing dressings or topical agents promote wound healing or prevent wound infection.

Silver nanoparticles (AgNPs) are known for their inherent antimicrobial properties and are now used extensively in bandages, clothing, and other materials to inhibit bacterial growth, including MRSA. Curiously, several laboratories have recently reported the ability of some microorganisms to produce antimicrobially active silver-containing protein nanoparticles biosynthetically, in the presence of AgNO_3_. One of these is a marine fungus, *Penicillium fellutanum*, isolated from coastal mangrove sediment (Kathiresan et al., 2009). A second report describes the isolation and antibacterial and antifungal properties of silver-containing nanoparticles 5–20 nm in size from callus and leaf extracts from coastal salt marsh species *Sesuvium portulacastrum L*. (Nabikhan et al., 2010)

Recently, silver ion was introduced into the protein apoferritin to form a stable Ag(I) complex, which was then assayed for *in vitro* activity against MRSA (Dospivova et al., 2012). The authors showed that the silver ions remain enclosed in the apoferritin structure until bacterial enzymes decompose the apoferritin scaffold.

**Figure 10:**
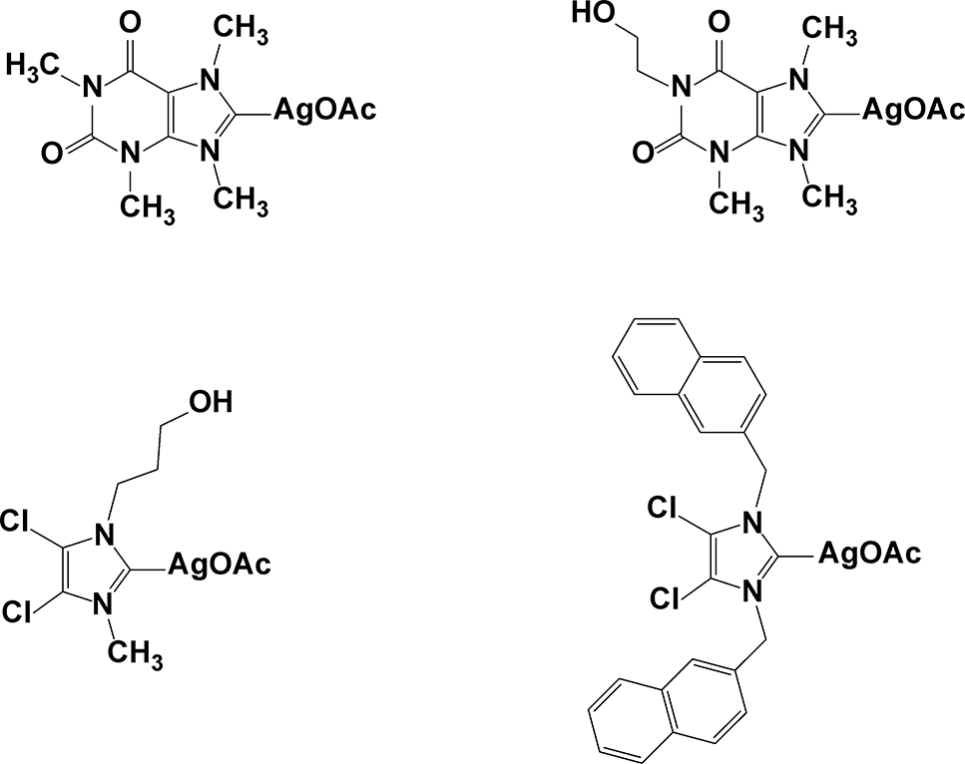
Silver carbene complexes.

More recently, Leid et al (Leid et al., 2012) described the *in vitro* anti-MRSA activity of silver carbene–based nanoparticles, which may represent novel, broad-spectrum antimicrobial agents with low toxicity (**Figure 10**). PLA and L-tyrosine polyphosphate were used as biodegradable polymers to efficiently entrap one of four silver carbene complexes. All four complexes on their own showed activity against a clinical isolate of MRSA, but their nanoparticle formulations were even more effective, with the most active one being the PLA nanoparticles, with an *in vitro* MIC of 1 µg/ml.

**Figure 11:**
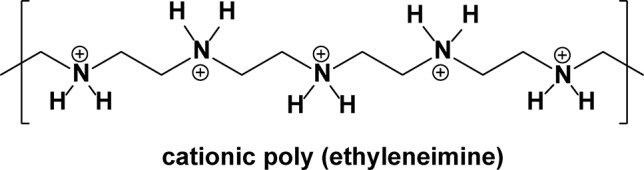
Structure of protonated poly(ethyleneimine).

In an alternative attempt to reverse *mecA*-dependent antibiotic resistance in MRSA, Meng et al. (Meng et al., 2009) have developed an anionic liposome for encapsulating and delivering the complexes of an anti-*mecA* phosphorothioate oligodeoxynucleotide (PS-ODN833) bound to a polycationic poly(ethyleneimine) (PEI) (**Figure 11**).

This offers a novel means for directly inhibiting *mecA* expression in MRSA, and therefore, restoration of susceptibility to β-lactam antibiotics. Effective antisense inhibition in cells requires an efficient mechanism of entry of the antisense agent into the bacterial cell, which is inherently a difficult problem to overcome for large, highly charged, hydrophilic molecules. However, these liposomes (measuring about 80 nm in diameter) can efficiently deliver the antisense oligonucleotide into bacteria to inhibit gene expression. The amount of encapsulated ODN833 within the liposomes was 16.4 μg/mg of total lipid. When implemented at the concentrations of 6 and 18 μM, the encapsulated PS-ODN833 nanoparticles reduced the *in vitro* MIC of oxacillin from 1,024 to 2 and 1 μg/ml, respectively, in the MRSA World Health Organization (WHO-2) strain, which are within the oxacillin sensitivity range for a non-resistant *S. aureus* strain. The *in vitro* expression of *mecA* messenger ribonucleic acid (mRNA) in the MRSA was measured by real-time polymerase chain reaction (PCR) and was not altered by treatment with either the empty liposomes, liposome-encapsulated mismatched PS-ODN, or free PEI. *In vivo*, the administration of encapsulated PS-ODN833 (at 2.5, 5, and 10 mg/kg) once daily by tail vein injection for 3 days, along with oxacillin (100 mg/kg) twice daily for 7 days, significantly improved the survival rate of septic mice, from 0% for the control group to 26.7%, 46.7%, and 53.3% for encapsulated-PS-ODN833–treated groups (in a dose-dependent manner). The findings of this study indicate that an antisense oligodeoxynucleotide targeted to *mecA* may be able to restore MRSA with susceptibility to existing β-lactam antibiotics.

Kilian et al. showed that liposome encapsulation of certain antibacterial agents such as amoxicillin may enhance activity as well as help overcome bacterial antibiotic resistance. In their study, the MIC of amoxicillin against MRSA was dramatically decreased from 2 to 0.0625 mg/ml (similar to that for methicillin-sensitive *Staphylococcus aureus* MSSA) and <0.008 mg/ml (against a second clinical isolate of MRSA), when it is encapsulated inside of liposomes at a 9.4% average (Kilian et al., 2011).

In 2007, our laboratory reported a simple procedure for preparing aqueous emulsions of antibacterially active polyacrylate nanoparticles, which enhance water solubility and *in vitro* antibacterial activity of highly lipophilic *N*-thiolated β-lactam antibacterials (Turos et al., 2007a). In this method, the water-insoluble antibiotic was first converted to an O- or N-acrylated derivative and then dissolved in a mixture of liquid monomers (butyl acrylate and styrene) before being emulsified with an anionic surfactant (sodium dodecyl sulfate, SDS) and polymerized in the presence of potassium persulfate (K_2_S_2_O_8_, a water-soluble radical initiator). This procedure affords a stable aqueous emulsion of polyacrylate–styrene nanoparticles, measuring uniformly around 45 nm in size and having *in vitro* antibacterial activity against MRSA (Garay-Jimenez et al., 2008; Garay-Jimenez et al., 2009). The polyacrylate–styrene nanoparticles have also been demonstrated to rejuvenate antibacterial activity of water-soluble penicillins against β-lactamase–producing MRSA strains (Turos et al., 2007b). Penicillin-containing polyacrylate nanoparticles, prepared by free radical emulsion polymerization in water using either acrylated penicillin monomers (for covalent attachment to the nanoparticle matrix) or non-acrylated penicillin esters (for encapsulation into the nanoparticle), retain their full antimicrobial properties even in the presence of ultrahigh concentrations of added β-lactamase. Moreover, the *in vitro* bioactivity of these “nanopenicillins” depends on the structure of the penicillin derivative used, as well as on the type of functionality linking the penicillin to the polymeric framework. The order of *in vitro* bioactivity observed in the assays follows in parallel the hydrolytic sensitivity of the chemical linker, with imide > ester > amide. As a consequence of the emulsion polymerization and high lipophilicity of the lactams, effectively all of the antibiotic (up to 5 weight %) is covalently attached to the polymeric matrix of the nanoparticle, which releases the active drug most likely by enzymatic hydrolysis of the linkage. A follow-up *in vivo* study of the penicillin-conjugated nanoparticle emulsions was carried out to evaluate toxicological responses during either systemic or topical application in a murine model. These showed favorable results for both routes of administration, suggesting that polyacrylate nanoparticle–containing emulsions may afford promising opportunities for treating both skin and systemic infections (Greenhalgh and Turos, 2009; Garay-Jimenez and Turos, 2011).

We have also described a method for synthesizing glycosylated poly(butyl acrylate–styrene) nanoparticles as antibiotic delivery vehicles for either water-insoluble or water-soluble penicillase-sensitive β-lactams. In either instance, the antibacterial agent can be attached to the nanoparticle framework through cleavable ester or imido linkages or simply encapsulated non-covalently into the polymeric matrix. This illustrates the possibility of employing relatively large, chemically sensitive acrylated monomers and carbohydrate motifs for the formation of emulsified polyacrylate nanoparticles and their utility against MRSA. This report was soon followed up with an additional advance, in the synthesis of glycosylated nanoparticles having ciprofloxacin attached to the matrix through an ester linker (Abeylath and Turos, 2007; Abeylath et al., 2008). Ciprofloxacin is a semi-synthetic analogue of the fluoroquinolone drug class that kills bacteria by interfering intracellularly with DNA gyrase and topoisomerase IV, the enzymes responsible for rewinding bacterial DNA following replication. As for the aforementioned β-lactam nanoparticle systems, strong anti-MRSA activity was observed during the preliminary *in vitro* testing of these ciprofloxacin nanoparticles, with MIC values of 32 mg/ml against both MSSA and MRSA strains.

In 2010, Durairaj et al. described a dendrimeric delivery vehicle for a different fluoroquinolone analogue, gatifloxacin, which is approved for treatment of bacterial conjunctivitis in the eye (Durairaj et al., 2010). In this study, guanidinylated dendrimers formed stable, ionic complexes with gatifloxacin, measuring about 350 nm in size. The dendrimer complex may be suitable for improving drug delivery to the eye, given that it could enhance the water solubility of the antibiotic fourfold and rapidly enter human corneal epithelial cells (within 5 min). Furthermore, the dendrimer gatifloxacin complex killed MRSA three times faster compared to gatifloxacin alone, achieved higher drug concentrations in the target tissues after *in vivo* delivery (in albino rabbits), and maintained satisfactory drug levels for 24 h, potentially allowing a once-daily dose regimen.

Fatty acids are endogenous bactericides that non-specifically disrupt bacterial membranes and increase membrane permeability. As such, they hold great promise as potential drug delivery vehicles while minimizing the onset of acquired resistance. In 2011, a study was conducted to evaluate the *in vitro* and *in vivo* anti-MRSA activity of oleic acid used as a liposomal formulation (Huang et al., 2011a). The liposomes measured about 80 nm in diameter. Interestingly, the liposomes were about 12-fold more effective than oleic acid. This enhanced activity is postulated to occur by fusion of the liposomes with bacterial membranes, leading to a “burst” release of a lethal dose of oleic acid into the intracellular environment, killing the bacteria.

A murine model was used to evaluate *in vivo* effectiveness of the liposomes in treating an MRSA skin infection. In this case, a single application of 100 μl of the liposome (at a concentration of 200 μg/ml) was effective at curing the skin infection within 48 h. Skin toxicity studies also showed high biocompatibility of the oleic acid liposomes with healthy skin tissue.

In a related study, albeit well beyond the dimensional boundaries of nanoparticles, microparticles measuring 5,300 nm prepared from a cyanobacteria strain of the order *Nostocales* (Bio33-Maresome™) could effectively inhibit colonization of different MRSA strains on human skin (Lukowski et al., 2008). These microparticles contain lipids and other cellular components that manage to induce stronger anti-colonization activity against MRSA than other bacteria making up the normal skin flora. The authors postulate that this differential activity is due to stronger extracellular lipase activity of *S. aureus*, which could lead to the release of antimicrobial fatty acids encapsulated inside the microparticles.

In 2009, the Friedman group reported the use of nanoparticles for the delivery of nitric oxide (NO) to bacterial cells (Martinez et al., 2009). NO is a lipophilic, naturally occurring free radical with a short half-life and high reactivity, able to easily pass through cell membranes. Endogenous NO is biosynthesized enzymatically in cells by nitric oxide synthase (NOS), using arginine as its substrate, but NO can also be synthesized non-enzymatically by the reduction of nitrite and nitrosothiols. NO modulates cellular cytokines involved in wound healing and has antimicrobial activity by disabling zinc metalloproteins during DNA replication and cell respiration. In the Friedman study, nanoparticles composed of silane hydrogels were used to encase NO within a dry matrix that releases NO upon being exposed to moisture. These nanoparticles were tested in a murine skin infection model by applying the powdered NO-encased nanoparticles directly onto open skin wounds infected with MRSA (Han et al., 2009). The authors observed reduced bacterial load and an acceleration of wound closure for the mice treated with the NO-releasing nanoparticles, compared to the untreated mice. To extend these findings, both topical and intralesional administration of the dry NO nanoparticles to an MRSA intramuscular murine abscess model showed an accelerated clearance of the abscesses 4 and 7 days later and a significant decrease in bacterial survival based on colony forming unit assays. These data suggest that the NO-releasing nanoparticles may be effective for MRSA soft tissue infections (Schairer et al., 2012). Furthermore, *S*-nitrosoglutathione, a potent NO donor formed by the reaction of NO with intracellular glutathione, is considered one of the strongest naturally occurring nitrosating agents. The efficiency of the NO-releasing nanoparticles in producing *S*-nitrosoglutathione in the presence of glutathione was examined, along with the subsequent *in vitro* anti-MRSA activity. When the nanoparticles were treated with glutathione, *S*-nitrosoglutathione was quickly produced, and significant concentrations were maintained for more than a day. The *S*-nitrosoglutathione generated served as a more effective antimicrobial agent than NO alone, consistent with the function of *S*-nitrosoglutathione as a cellular nitrosating agent. Even more importantly, *S*-nitrosoglutathione used at similar concentrations to those generated from the nanoparticles did not impede bacterial growth or cell survival (Friedman et al., 2011).

Although the mechanism for how the nanoparticles interact with bacterial cells is not fully understood, it seems likely that they may fuse to the lipid bilayer of microbial cells to release the drugs within the cell wall or at the membrane. In this way, the nanoparticles may serve as a drug depository to continuously release the antibiotic at the surface of cells and subsequently diffuse into the microorganisms. Alternatively, some drug-loaded nanoparticles may be able to enter cells *via* endocytosis prior to releasing the drug payload.

## Nanoparticles as Anti-MRSA Agents

**Figure 12:**
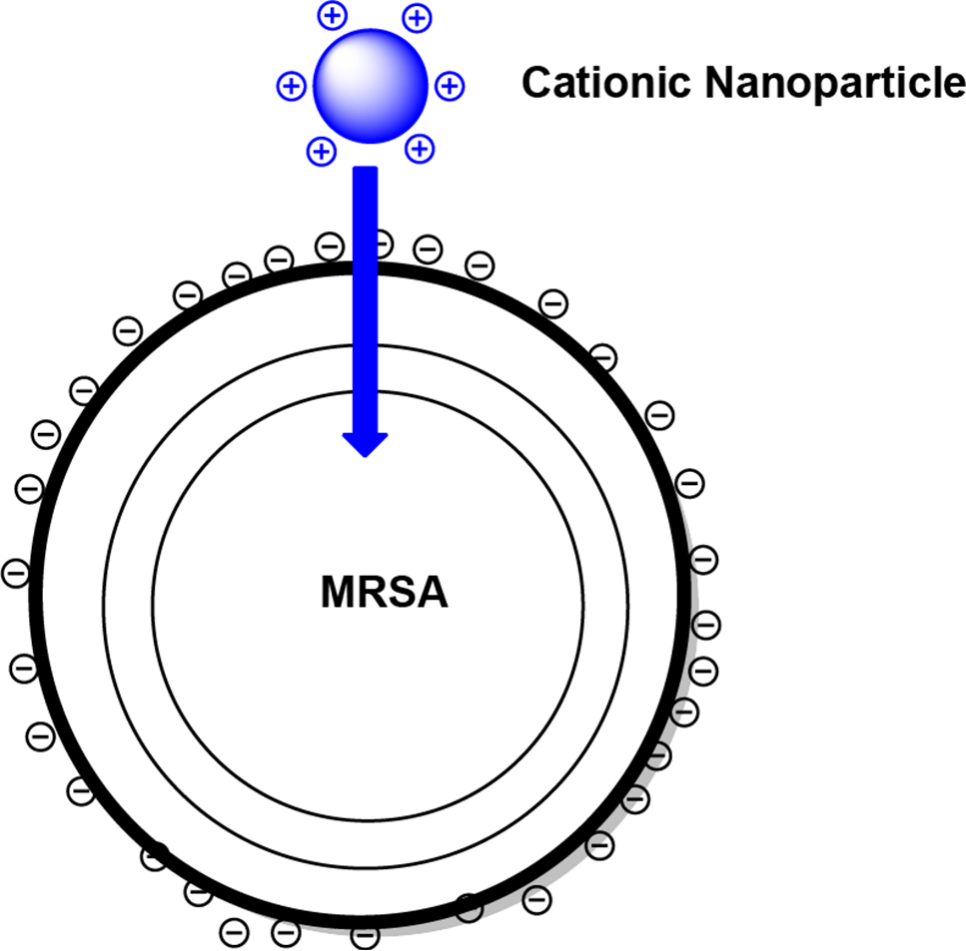
A schematic representation of cationically charged nanoparticles interacting with a methicillin-resistant *Staphylococcus aureus* (MRSA) cell.

Apart from the nanoparticles developed explicitly for the delivery of anti-MRSA agents, the use of nanoparticles as anti-MRSA agents on their own has also been explored to some degree. In these instances, the choice of material used to construct the nanoparticle is of critical importance. Several types of constructs have been investigated for anti-MRSA applications. Starting first from the biological side, the broad-spectrum antimicrobial activity of naturally occurring cationic peptides affords potential advantages against multi-drug-resistant microbes. The unique propensity of these peptides to insert into lipid bilayers enables them to intimately meld into the target bacterium, while disrupting the integrity and function of bacterial cell membranes. Because of similar attributes, cationically charged nanoparticles possess inherent antimicrobial activity, wherein the high concentration of charge localized on the nanoparticle may be deposited from the presence of amines (protonated at physiological pH) or tetraalkyl ammonium groups protruding from the outer surface, ionically interacting with the negatively charged bacterial membrane surface (**Figure 12**) (Engler et al., 2012).

**Figure 13:**
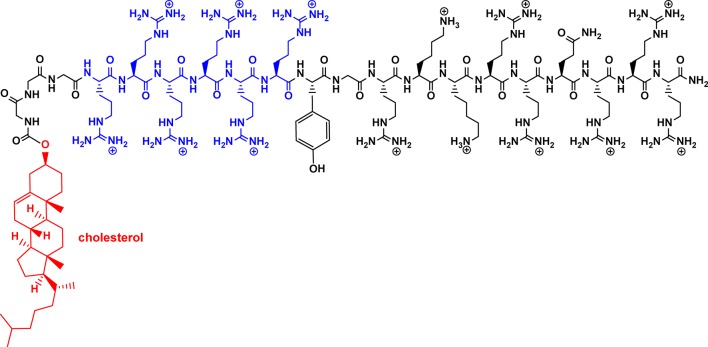
Structure of an amphiphilic peptide.

The Yang group reported amphiphilic peptides that self-assemble into a core–shell nanoparticle assembly (Liu et al., 2009). These peptides are comprised of a *hydrophilic* peptide fragment TAT (YGRKKRRQRRR) containing six arginine residues (shown below in blue) providing cationic charges required for disrupting the membrane, as well as a *hydrophobic* unit of cholesterol, separated from the rest of the peptide chain by three glycines (**Figure 13**).

These designed oligopeptides self-assemble into micelles measuring under 150 nm in size that enhance the local concentration of cationic charge on the outer surface, and along with that, antimicrobial activity. Bioassays indicated that the peptidic nanoparticles have *in vitro* activity against MRSA, with an MIC of 11.4 µM, which equates to approximately 35 µg/ml. Compared to the non-associated peptides, the nanoparticle micelles showed higher therapeutic efficacy in treating *S. aureus* infection in mice and presumably would perform as well toward MRSA, which unfortunately was not investigated. Furthermore, in rabbits, the nanoparticles administered intraperitoneally were able to cross through the blood–brain barrier to suppress bacterial growth in *S. aureus*–infected brains.

**Figure 14:**
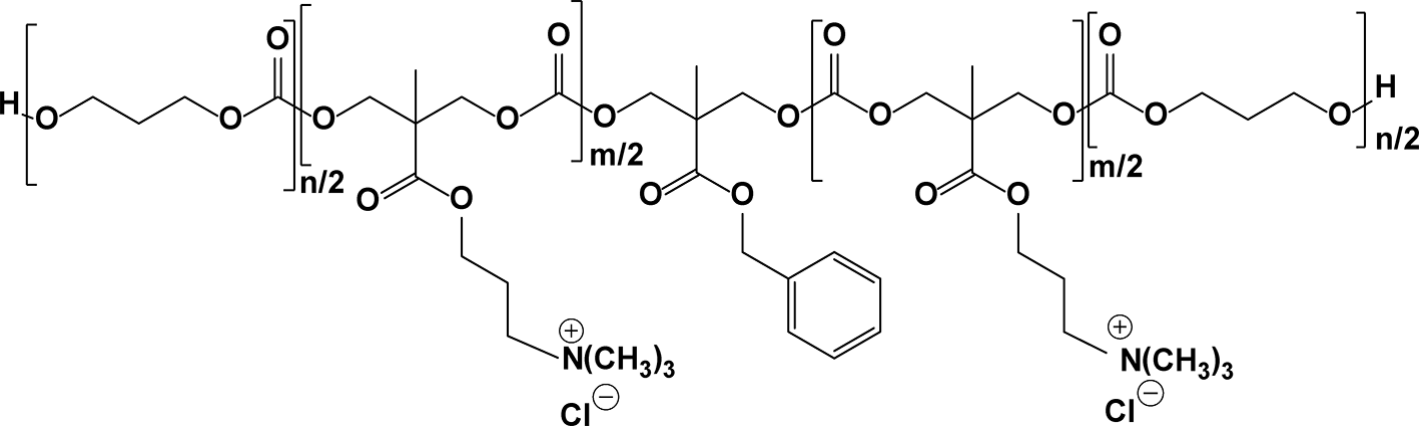
Structure of an amphiphilic polycarbonate.

This group also described cationic, amphiphilic polycarbonates that are biodegradable, which, like those of the oligopeptides above, also self-assemble into micelles in water (**Figure 14**) (Nederberg et al., 2011). *In vitro* testing confirms that these nanoparticles can efficiently kill MRSA (MICs ∼ 30–60 µg/ml depending on the tested polymer). No significant hemolytic activity or acute toxicity to the major organs in non-infected mice was observed.

**Figure 15:**
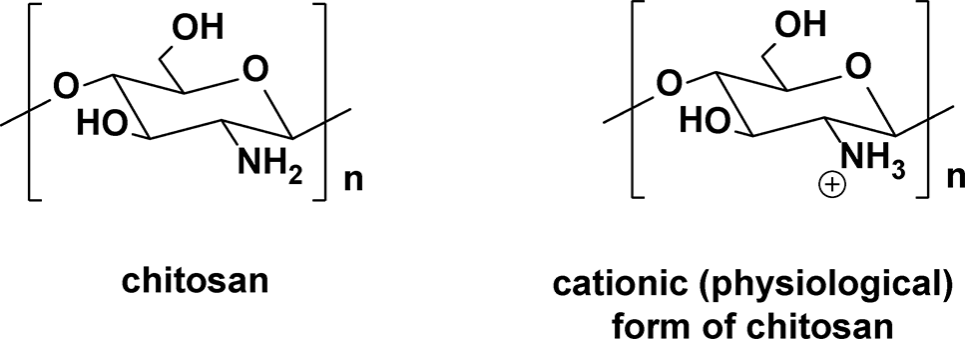
Chitosan and its cationic (physiological) protonated form.

Dugal and Mamajiwala (2011) investigated the anti-MRSA properties of chitosan nanoparticles having quaternary ammonium groups on the surface, prepared by ionic gelation (**Figure 15**). Chitosan is a polysaccharide of (Crossley et al., 2009; Tong et al., 2015)-β-linked D-2-glucosamines, which have strong antimicrobial properties. Chitosan is considered to be biocompatible and biodegradable. The ammoniated chitosan nanoparticles average around 100 nm in diameter, as determined by dynamic light scattering, with a high zeta potential of +88 mV. Assays indicate that they have potent *in vitro* activity against MRSA biofilms, exceeding that of the non-ammonium cationic chitosan nanoparticles (MICs = 3,000 µg/ml for the chitosan nanoparticles compared to 400 µg/ml for the corresponding quaternary nanoparticles). The authors attribute the enhanced anti-MRSA activity to the polycationic charges on the surface of these nanoparticles that are presumed to interact with the negatively charged surface of the bacterial cell, weakening the structural integrity of the membrane.

Metal nanoparticles have likewise been widely examined as anti-infective agents, with silver nanoparticles being the most common (Rai et al., 2009; Sharma et al., 2009; Chaloupka et al., 2010; Pissuwan et al., 2010; Wong and Liu, 2010; Samberg et al., 2011; Guzman et al., 2012; Vigderman and Zubarev, 2013; Li et al., 2014; Durán et al., 2016) Silver nanoparticles, in addition to being used in nanoparticle composites with other materials, can be used on their own as metal nanoparticles. They are perhaps one of the most widely studied types of nanoparticle for antibacterial purposes. Silver is a particularly good antibacterial agent because it can be taken up by bacterial cells and traverse through cell walls (Feng et al., 2000). Moritz et al. describes how silver ions are produced on the surface of some nanoparticles and can change the permeability of the cell membrane (Moritz and Geszke-Moritz, 2013). Silver ions also have interactions with thiol groups on cysteine (Liau et al., 1997) and disrupt bacterial DNA replication (Feng et al., 2000) because silver has a strong affinity for phosphorus centers in the DNA backbone.

The cellular mechanism for antimicrobial action of silver nanoparticles has not been completely defined but may be similar to that of bulk silver and enhanced by having much greater surface area, which enables more favorable association with and penetration inside bacteria cells. In addition to targeting proteins in the membrane and cytoplasm, silver nanoparticles can attack the respiratory chain and lead to cell death. Silver nanoparticles could also provide sustained release of Ag^+^ inside bacterial cells to induce a constant level of oxidative stress, as a means for enhancing bactericidal activity (Panacek et al., 2006; Yu, 2007; Kvítek et al., 2008; Huang et al., 2009; Huang et al., 2010; Le et al., 2010; Stevanović et al., 2012). Silver nanoparticles can be made in a multitude of ways, by chemical, photochemical, or electrochemical reduction of Ag^+^ ion in aqueous solution or by ultrasonic dispersion of elemental silver in water. The dimensions and shape of the final nanoparticles may be further controlled by the use of surface-bound stabilizing agents such as surfactants. Within the last decade, there has been increasing interest to implement environmentally more friendly methods for producing metal nanoparticles, such as the use of non-toxic chemicals and solvents and renewable materials. Microbiological methods have been examined using bacteria and fungi as the growth media for producing metal nanoparticles. Several laboratories have recently reported the ability of some microorganisms to produce antimicrobially active silver-containing protein nanoparticles biosynthetically, in the presence of AgNO_3_. One of these is a marine fungus, *P. fellutanum*, isolated from coastal mangrove sediment (Kathiresan et al., 2009). A second report described the isolation and antimicrobial (bacterial and fungal) properties of silver-containing nanoparticles from callus and leaf extracts of the coastal salt marsh species *S. portulacastrum L*. (Nabikhan et al., 2010)

**Figure 16:**
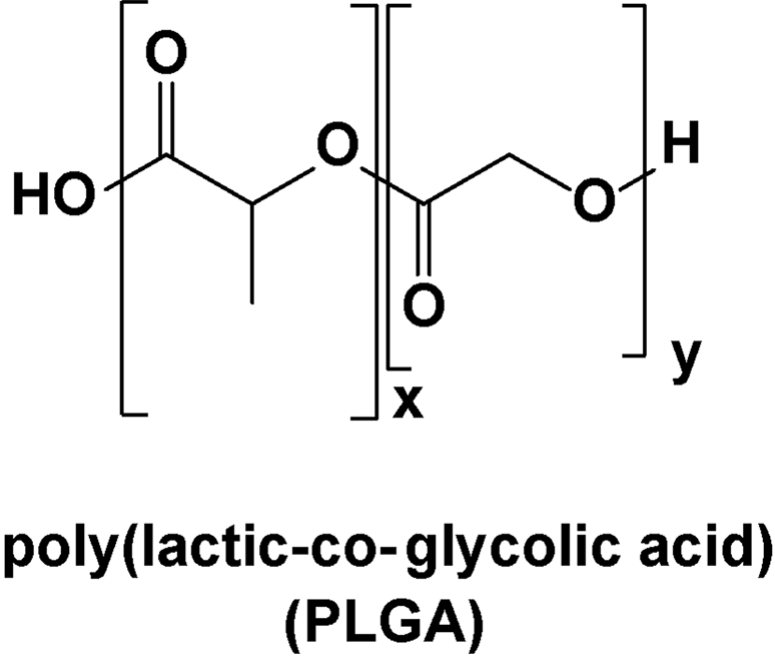
Structure of poly(lactic-co-glycolic acid) (PLGA).

Several groups have synthesized silver nanoparticles by chemical means and evaluated their *in vitro* properties against MRSA. Silver nanoparticles stabilized with poly(ခγ-glutamic acid), prepared by *in situ* reduction of Ag(I) using dextrose, formed in sizes of 15–60 nm(115). Similarly, silver nanoparticles prepared using a sucrose ester micelle-mediated approach led to diameters in the range of 10–25 nm (Huang et al., 2010), or the introduction of chitosan as a stabilizing agent under γ-irradiation (Huang et al., 2009) gave sizes in the 5–25 nm range as well as high anti-MRSA activity (MIC = 100 ppm). Colloidal silver particles can be synthesized in water by reduction of [Ag(NH_3_)_2_]^+^ with common reducing sugars, such as glucose, galactose, maltose, and lactose. The reduction of [Ag(NH_3_)_2_]^+^ by maltose produced silver nanoparticles with an average size of 25 nm (Panacek et al., 2006). They were found to be more active against MRSA when the surface was modified by an anionic surfactant such as SDS (MICs = 1.69 µg/ml compared to 3.38 µg/ml for the uncoated nanoparticles) (Kvítek et al., 2008). It was also shown that silver nanoparticles released from PLGA microparticles were more active against MRSA *in vitro* than the bare silver nanoparticles (MICs ∼ 0.005 µg/ml compared to 0.24 µg/ml for the non-encapsulated nanoparticles) (**Figure 16**) (Stevanović et al., 2012).

Different silver nanoparticles obtained by photochemical synthesis (Le et al., 2010) or by biosynthesis (Nanda and Saravanan, 2009; Saravanan and Nanda, 2010; Raghunandan et al., 2011) also have shown anti-MRSA activity *in vitro*. Using commercially available silver nanoparticles, good anti-MRSA activities were obtained (MIC ∼ 10 µg/ml), and similar results were also observed on methicillin-sensitive *S. aureus*, highlighting the non-specific antibacterial effect of silver nanoparticles (Ayala-Núñez et al., 2009; Rai et al., 2009; Samberg et al., 2010; Ansari et al., 2011; Kvitek et al., 2011; Brandt et al., 2012). It was also demonstrated that the size of silver nanoparticles influences the anti-MRSA activity and cytotoxicity toward human cells. The smaller nanoparticles (10 nm) were very active against MRSA while displaying no cytotoxicity. However, 100 nm nanoparticles were cidal against both the bacteria and the eukaryotic cells. Silver nanoparticles are also much less toxic than ionic silver against eukaryotic cells. This seems to indicate that a high surface-to-volume ratio of the silver nanoparticles ensures an inherent selectivity for the bacteria cell, perhaps due to easier penetration through the membrane to gain entry into the cell. The antibacterial activity of ionic silver is correlated to its valence state, in that high-valency silver ion Ag(III) exhibits stronger antibacterial properties than Ag(I) (Djokić, 2004). The antimicrobial effectiveness of a smaller-particle-size silver sulfadiazine product and stabilization of these higher-valent silver particles by adsorbed surfactant prompted Pal et al. (2009) to develop silver(III) polydiguanide nanoparticles synthesized in a reverse microemulsion. The wet chemical preparation of silver(III) polydiguanide can be realized by oxidizing a mixture of a biguanide base or its salt and silver nitrate solution in feebly acidic or neutral medium. However, this preparative method does not allow strict control over the size of the final crystalline product. The antibacterial activity of the nanoparticles of a silver(III) complex was ascertained *in vitro* against an MRSA strain, and the MICs of the nanocrystalline Ag(III) complex were 30 times lower than those of AgNO_3_. Because of their ambient stability and high monodispersity, these nanoparticles are expected to improve the homogeneous and uniform distribution of the active agent in antibacterial creams or wound dressings.

**Figure 17:**
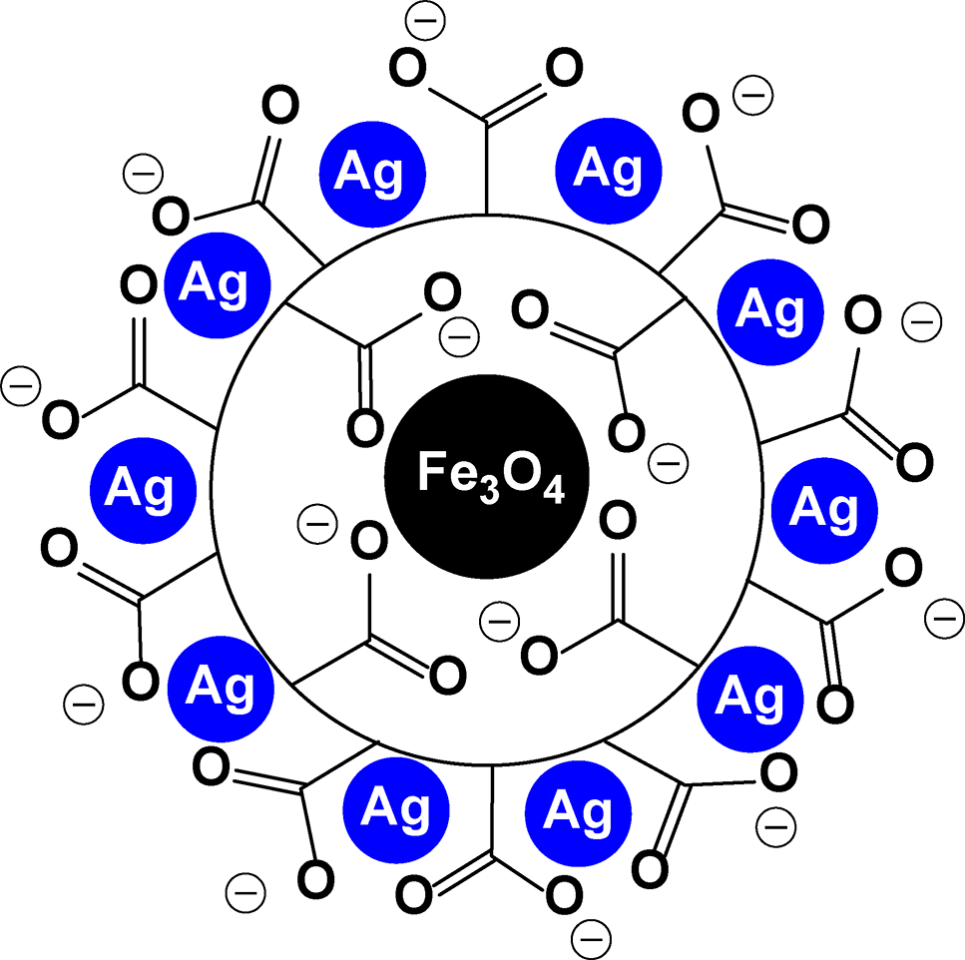
A schematic representation of a binary silver-coated iron nanoparticle.

The Zboril group has reported the synthesis and anti-MRSA activity of binary composites made of silver and iron nanoparticles (**Figure 17**) (Dallas et al., 2011; Prucek et al., 2011). Polyacrylate was used as a surface matrix between the silver and iron core nanoparticles.

Recently, copper and copper oxide (CuO) nanoparticles have also been shown to be effective in killing MRSA *in vitro* (Ren et al., 2009). Compared to silver nanoparticles, however, higher concentrations were required to achieve a bactericidal effect. The mechanism of action of copper nanoparticles is thought to be analogous to that of silver nanoparticles, in this case, by release of Cu^2+^ ions that cause local pH and conductivity changes that somehow disrupt bacterial cell membranes and alter the function of key intracellular targets such as respiratory enzymes.

Correspondingly, zinc oxide (ZnO) nanoparticles have been investigated against clinical isolates of MRSA and MSSA *in vitro* but found to be less effective than either copper or silver nanoparticles (Jones et al., 2008; Ansari et al., 2012). ZnO nanoparticles are presumed to cause cell lysis by damaging lipids and proteins in the bacterial cell membrane, thereby leading to the leakage of cytosolic material. It has also been suggested that the ZnO nanoparticles cause the formation of H_2_O_2_, which is lethal to bacteria. The use of high concentrations of ZnO nanoparticles (5 mM) inhibits the growth of MRSA, and this anti-MRSA activity of the nanoparticles was mainly found after their photoactivation under ambient light conditions, and to a slightly lesser extent, in the absence of light as well. Optimal bioactivity was observed when the smallest size of nanoparticles was tested (Raghupathi et al., 2011).

Titanium oxide (TiO_2_) nanoparticles have also been investigated for anti-MRSA activity (Roy et al., 2010). The combined effect of TiO_2_ nanoparticles with a wide range of different antibiotics (cephalosporins, glycopeptides, aminoglycosides, fluoroquinolones, azalides, macrolides, lincosamides, sulfonamides) was also evaluated for synergistic anti-MRSA activity by a disk diffusion assay. The most significant synergic effect was observed in the case of penicillin G. Under ultraviolet (UV) photoactivation, TiO_2_ nanoparticles are postulated to form OH radical, superoxide, and H_2_O_2_. These photoactivated TiO_2_ nanoparticles showed anti-MRSA activity (Dallas et al., 2011).

These different metal nanoparticles may also be utilized as anti-infective surface coatings on medical devices and surgical implants (Alt et al., 2004; Inoue et al., 2009; Necula et al., 2009; Edmiston and Markina, 2010; Maki, 2010; Zheng et al., 2010; Nirmala et al., 2011; Stevens et al., 2011; Engler et al., 2012) Most commonly, silver nanoparticles are popular as wound-dressing materials, (148, Rujitanaroj et al., 2008; Lee et al., 2010) and surface coatings on equipment in health care facilities (Stobie et al., 2009; Su et al., 2009; Dallas et al., 2010; Holtz et al., 2010; Shameli et al., 2010; Dunnill et al., 2011; Shameli et al., 2011a; Shameli et al., 2011b; Su et al., 2011; Holtz et al., 2012; Rathnayake et al., 2012; Shastri et al., 2012). Copper, (Bagchi et al., 2012) ZnO, (Perelshtein et al., 2012) and TiO_2_ nanoparticles (Kangwansupamonkon et al., 2009) provide especially effective anti-MRSA coatings.

Antimicrobial coatings based on a carbon nanotube–enzyme conjugate have also been investigated as anti-infective materials against MRSA. The conjugation of carbon nanotubes with lysostaphin, a cell wall–degrading enzyme, produces films as a means to prevent growth of pathogenic and antibiotic-resistant microorganisms on various surfaces in hospitals (Pangule et al., 2010).

## Nanoparticle Combinations

A combination of the strategies described above has been implemented through several approaches. The decoration of nanoparticles with anti-MRSA agents showed a synergistic antimicrobial effect *in vitro*. Furthermore, anti-MRSA nanoparticles have been used for photodynamic therapy, such as liposomes for photosensitizer delivery and photosensitizers covalently bound to inorganic nanoparticles. As an example, the combination of gold nanoparticles with a photosensitizing agent has been reported to afford a combination photodynamic inactivation through singlet oxygen formation and a cytotoxic hyperthermia effect. Conversely, the appending of certain cellular targeting moieties such as antibodies to the surface of the nanoparticle can enhance selective binding to either microorganisms or infected cells, to permit the controlled release of the antibiotic.

### Nanoparticle/Anti-Mrsa Agent Conjugates

In 2011, Huang, et al. (2011b) demonstrated the synergistic advantage of combining chitosan acetate and silver nanoparticles to prepare antimicrobial burn dressings. The material was shown to be effective in an *in vivo* burn model in mice infected with *Pseudomonas aeruginosa*, and although not investigated against MRSA, the dressing may also improve permeabilization of bacteria and penetration of silver ion into tissue. The authors showed that the chitosan–silver nanoparticles improved *in vitro* growth inhibition of MRSA.

Similarly, silver nanoparticles have been conjugated to antimicrobial peptides, and their *in vitro* microbiological properties, examined (Golubeva et al., 2011). Those bearing the 12-amino-acid antimicrobial peptide G-Bac3.4 provided anti-MRSA activity without a strong membrane lytic effect (as demonstrated on the model microorganism, *Escherichia coli*), typically associated with the cytotoxic properties of antimicrobial peptides. This conjugate was prepared in order to decrease the toxicity of the free peptide toward healthy tissues.

Silver nanoparticles have also been prepared with ampicillin, a β-lactam antibiotic, chemophysically adsorbed onto the surface (Brown et al., 2012). These nanoparticles showed a twofold increase in *in vitro* anti-MRSA activity relative to the silver nanoparticles alone.

**Figure 18:**
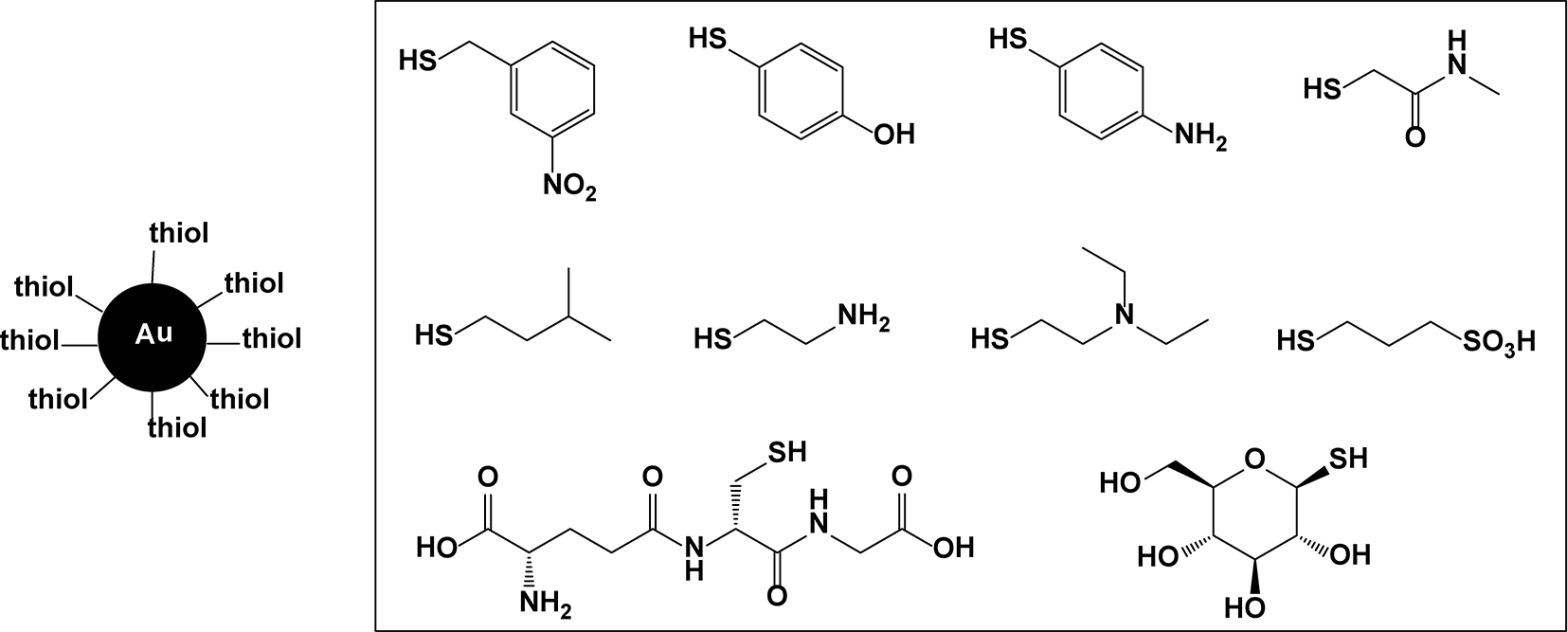
Thiols used to surface-coat gold nanoparticles.

A similar effect was found with ampicillin-bound gold nanoparticles as well. Additional ligand-coated gold nanoparticles having anti-MRSA properties have also been described in the literature, (Bresee et al., 2011) using thiol exchange reactions to append multiple types of organothiol ligands as monolayers on the gold surface (**Figure 18**). In this way, the binding affinity to a biological target and cellular internalization properties can be adjusted. A library of 95 different ligand-coated gold nanoparticles was tested for the inhibition of *in vitro* bacterial growth, with one particular conjugate showing 99.9% growth inhibition at 10 µM for both MSSA and MRSA. The mechanism by which this inhibitory effect exists is not known, but it may be attributable to improved cellular internalization and aggregation of the nanoparticles inside the bacterial cells, or conversely, to targeted binding to the cell surface.

### Photodynamic Therapy

Photodynamic therapy constitutes a well-established method for the treatment of a variety of solid tumors (Vanderesse et al., 2011). Operationally, a non-toxic photosensitizing agent is used to kill malignant cells by generating reactive oxygen species by absorbing light as a means for generating reactive oxygen species in or around the cancerous tissue. Similarly, the application of this method to kill bacteria in tissue may afford the advantage over traditional antibiotics of not selecting for resistant mutants. Recently, several groups have explored the implementation of light-activated nanoparticles for photodynamic antimicrobial therapy to enhance delivery of the photosensitizing agent to sites of bacterial infection or the efficacy of bacterial cell inactivation (Perni et al., 2011).

Among these explorations, liposomes have been investigated for delivery of selected photosensitizing agents. Non-cationic liposomal formulations had anti-MRSA activity comparable to that of the free photosensitizer (hematoporphyrin), (Ferro et al., 2006) while cationic variants were more efficient. An explanation is that the cationic liposomes can interact with and disrupt the bacterial membrane to allow for improved permeability of the photosensitizer into the membrane (Bombelli et al., 2008). Similarly, the combination of cationically charged photosensitizers with cationic liposomes induces strong anti-MRSA activity (Ferro et al., 2007). The use of photosensitizers coupled with cationic cyclodextrin nanoaggregates of amphiphilic β-cyclodextrin (mean diameter of 20 nm), (Ferro et al., 2009) polymeric micelles, (Tsai et al., 2009) and certain inorganic nanoparticles such as silica nanoparticles (Guo et al., 2010) effectively inhibit MRSA growth *in vitro*. These nanoparticle-based photosensitizers are more resistant toward photobleaching, and the generation of singlet oxygen at the nanoparticle surface rather than throughout the solution ensures a higher local concentration that is lethal to the microbe. Attachment of the photosensitizer to the surface of a gold nanoparticle is readily achieved *via* a glutathione linking group, and the conjugated nanoparticles afford anti-MRSA activity upon irradiation with white light for 20 min. (Gil-Tomás et al., 2011) Photosensitizers in combination with gold nanoparticles have also been implemented for coating surgical devices to reduce the survival of MRSA *in vitro* (Perni et al., 2009). Gold nanoparticles were also used as dual-function materials for combined photodynamic and hyperthermal killing of MRSA (Kuo et al., 2009). In the latter case, toluidine blue–conjugated gold nanorods exhibited 79% and 70% bacterial cell viability, respectively, after laser irradiation of culture plates at 633 nm (for photodynamic therapy) and 808 nm (for photothermolysis), but viability fell dramatically to 14% after sequential irradiation at 633 and 808 nm.

### Targeted Nanoparticles

Two basic requirements should be realized in the design of nanocarriers to achieve effective drug delivery. First, drugs should be able to reach the desired sites after administration with minimal loss to their volume and activity in blood circulation. Second, drugs should only kill targeted cells without harmful effects to healthy tissue. Targeted therapeutic nanoparticles that accumulate in a particular tissue have shown promising results. Recently, nanoparticles featuring a homing molecule that allows them to specifically attack cancer cells are the first such targeted particles to enter human clinical studies. Bacteria have remarkable capacities to develop resistance to antibiotics. However, much higher doses than usual of these antibiotics can still be effective, but it is normally not feasible to administer such high doses to patients due to the side effects these drugs have. Highly targeted nanoparticles that deliver huge doses of existing antibiotics could be used to overload the defenses of drug-resistant bacteria. In regard to the *S. aureus* infection, gold nanoparticles conjugated with anti–protein A antibodies have been examined for selective targeting and hyperthermal killing of the bacteria (Zharov et al., 2006). In this particular study, only *in vitro* activity against MSSA was demonstrated. Furthermore, MRSA photokilling was then achieved using photoactive iron oxide/titania (Fe_3_O_4_@TiO_2_) core/shell magnetic nanoparticles onto which immunoglobulin G (IgG) protein was grafted onto the surface (Chen et al., 2008). The IgG–nanoparticles were able to selectively target several pathogenic bacteria and inhibit cell growth *in vitro* under brief irradiation of a low-power UV lamp. Despite UV light transmission through tissue being limited, this approach may be applicable for the treatment of cutaneous bacterial infections. The nanoparticles have weaker interactions with MRSA than MSSA because Pls protein (plasmin-sensitive protein) on the surface of MRSA cells can sterically hinder the binding of protein A with IgG (Josefsson et al., 2005).

Liposomes with a mean diameter of ∼100 nm have been loaded with temoporfin, a potent photosensitizing agent, for photodynamic antimicrobial therapy (Yang et al., 2011; Yang et al., 2012). A first series was decorated with WLBU2, a 24-amino-acid antimicrobial oligopeptide found to have high antimicrobial activity toward *S. aureus*. WLBU2 binds effectively to various microbial species and may be a selective bacteria-targeting ligand. The WLBU2-bound liposomes exhibit enhanced toxicity to MRSA cells *in vitro* compared to the non-bound liposomes, at 1.25 μM of entrapped temoporfin after laser illumination (Yang et al., 2011). In a similar manner, bacteria-targeting liposomes loaded with temoporfin were obtained by conjugation of wheat germ agglutinin onto the surface of the liposomes (Yang et al., 2012). These agglutinins have high specificity for the components of peptidoglycan. Therefore, Gram-positive bacteria where the cell wall is directly exposed to the external medium bind more efficiently than Gram-negative bacteria. Flow cytometry measurements showed that surface modification with wheat germ agglutinin increased the delivery efficiency compared to the non-bound liposomes, and this delivery was more pronounced for MRSA than the only other strain tested, *P. aeruginosa*. *In vitro* photodynamic inactivation at 12.5 µM of entrapped temoporfin confirmed that these agglutinated liposomes may be useful for targeted killing of MRSA.

Another example is that of gold nanoparticles surface-bound with vancomycin, which, under irradiation of near-infrared light (808 nm), can induce the photothermal destruction of bacteria with high efficiency (Huang et al., 2007). The nanoparticles appear to be non-toxic to human cells.

Iron oxide (Fe_3_O_4_) nanoparticles conjugated with [5,15-bisphenyl-10,20-bis(4-methoxycarbonylphenyl)porphyrin]platinum, a photosensitizer, and vancomycin show good biocompatibility, with a capacity to selectively target and thermally destroy MRSA upon irradiation at 510 nm (Choi et al., 2012). The study found that the orientation and the mode of attaching the vancomycin onto the surface of the nanoparticles are important in order to maximize binding to bacterial cells.

Lastly, a new bacteria-targeting approach developed by Pornpattananangkul et al. (2011) utilizes bacterial toxins to promote drug release from gold nanoparticle–stabilized phospholipid liposomes. Vancomycin-loaded liposomes are protected by absorbing chitosan-coated gold nanoparticles (AuChi) onto their surface to prevent them from fusing with one another or with bacterial membranes. Once the AuChi-stabilized liposomes (AuChi–liposome) encounter bacterial toxins, the toxins form pores in the liposome membranes and thus release the encapsulated antibiotics, which subsequently kill or inhibit the growth of the bacteria that secrete the toxins.

The unique feature of these particular liposomes is that they can detect those bacteria that secrete protein toxins, which insert into the liposomal membrane to form pores through which the encapsulated antibacterial agent can be released. The authors first determined the optimum gold nanoparticle–to–liposome molar ratio and then optimized the formulation to maximize the highest pore-forming ability of the α-toxin by adjusting the cholesterol concentration in the liposomal membrane and the amount of poly(ethylene glycol). Both additives have previously been reported to affect the pore-forming activity of toxins in artificial membranes. With MRSA as a model bacterium and vancomycin as a representative anti-MRSA agent, the authors showed that the gold nanoparticle–stabilized liposomes can completely release the encapsulated vancomycin within 24 h in the presence of MRSA. Vancomycin’s MIC against MRSA is about 2 μg/ml, and the concentration of vancomycin released from the liposome was as high as 62 μg/ml, sufficient to inhibit MRSA growth. This may provide for an effective approach for the treatment of infections caused by not only MRSA but also other pathogenic bacteria that similarly secrete pore-forming toxins.

## Toxicological Considerations

An important aspect of nanoparticle-based therapeutics pertains to their potential cellular and systemic toxicity, which, in the general sense, remains a rather under-investigated realm of nanoscience. Early investigations by Kreuter related nanoparticle size to therapeutic capabilities and metabolic lifetime (Kreuter, 1991). It is now commonly appreciated that large nanoparticles (diameters of several hundred nanometers) and microparticles can be easily filtered from vasculature by lung capillaries and rapidly cleared by the reticuloendothelial system, while nanoparticles under 100 nm in gross dimensions can likely pass rapidly through fenestrations in the liver endothelium and the sieve plates of sinusoids, to ultimately localize in the spleen and bone marrow (Yoshioka et al., 1981; Jaspreet et al., 2005; Mikklesen et al., 2011; You et al., 2014)

A wealth of information has been gathered on the cellular toxicity and genotoxicity, biodistribution, and systemic toxicity of silver nanoparticles in animal models, as a function of particle sizes and route of exposure (oral, respiratory, transdermal, etc.). Despite the unknown, some silver wound dressings can be found commercially available in drug stores and supermarkets. In other applications, silver nanoparticles may cause lung inflammation in rodents with certain genes (Scoville et al., 2017). These findings might translate to genetic predisposition for human patients to develop lung inflammation when exposed to silver nanoparticles by various methods. Interested readers are directed to the cited references (Yoshioka et al., 1981; Kreuter, 1991; Jaspreet et al., 2005; Kawata et al., 2009; Sung et al., 2009; Tang et al., 2009; Kim et al., 2010; Lankveld et al., 2010; Lu et al., 2010; Hackenberg et al., 2011; Mikklesen et al., 2011; Park et al., 2011a; Park et al., 2011b; Park et al., 2011c; Kang et al., 2012; Singh and Ramarao, 2012; Kim et al., 2013; Park, 2013; Quang Huy et al., 2013; Suliman et al., 2013; Elkhawass et al., 2014; Gliga et al., 2014; Majdalawieh et al., 2014; You et al., 2014; Gao et al., 2017; Scoville et al., 2017) for specific toxicity studies that have been conducted on nanoparticles. Similar investigations on humans, of course, have been restricted, and thus, the background information is lacking. Other types of nanoparticles, in particular, inert metal nanoparticles, may be assumed to have similar properties and should be used with appropriate caution. Minimizing or removing undesirable side effects of any nanoparticle drug or drug carrier (associated with the unique particle size, material, and metabolic alterations) is a critical objective when developing potential biomedical applications.

It is worth pointing out as well that despite being composed of inert, biodegradable, or otherwise non-toxic materials, the nanoparticle forms may not be at all innocuous within a cell or living system. Aggregation phenomena such as agglutination or precipitation within blood vessels or membranes, or concentration within fat cells, bone tissue, or major organs, can be potentially problematic and even lethal. Therefore, it would be prudent to proceed to consider the nanoparticles to be a potential hazardous material to be handled with extreme caution, especially with regard to their drug delivery applications, until an accumulated body of knowledge about their short-term and long-term health effects is available. **Table 1** provides a generalized listing of some of the toxicological effects for different types of nanoparticles, with suitable lead references in addition to those in the references section.

**Table 1:** Summary of toxicological effects of different nanoparticles.

Types of nanoparticles	Toxicological effect	References
PLGA	Generally low cytotoxicity. No inflammatory response in mice.	Makadia, H.K., and Siegel, S.J. *Polymers (Basel)* 2011, *3*, 1377–97 Aragao-Santiago, L. et al. *Nanotoxicology* 2016, *10*, 292–302
Polycaprolactone	No cytotoxicity or histopathological lesions in mice.	Bansal, V. et al. *Vaccine* 2015, *33*, 5623–32
Polyacrylate	No inflammation and cytotoxicity after intranasal or topical administration in a murine model.	Ren, H., and Huang, X. *European Respiratory Journal* 2010, *36*, 218–21 Turos, E. et al. *Nanomedicine* 2009, *5*, 46–54
Liposomal	No side effects.	Huwyler, J., Drewe, J., Krahenbuhl, S. *International Journal of Nanomedicine* 2008, 3, 21–9
Chitosan	Induce malformations, cytotoxicity, and physiological stress in zebra fish embryos. Toxic to mice embryos.	Hu, Y.L. et al. *International Journal of Nanomedicine* 2011, *6*, 3351–9 Park, M.-R. et al. *Biology of Reproduction* 2013, *88*, 1–13
Silica	Moderate toxicity to lungs, kidneys, liver, and brain depending on the route of administration in mice.	Murugadoss, S. et al. *Archives of Toxicology* 2017, *91*, 2967–3010
Silver	Histopathological lesions and cytotoxicity in mice. Inflammatory response and accumulation of the nanoparticles in several tissues in rats.	Cho, Y.-M. et al. *Journal of Toxicological Pathology* 2018, *31*, 73–80 Recordati, C. et al. *Particle and Fibre Toxicology* 2016, *13*, article number 12
Gold	Inflammatory response in mice and rats.	Bahamonde, J. et al. *Toxicologic Pathology* 2018, *46*, 431–43
Copper	Important toxicity toward kidney, liver, and spleen in mice.	Chen, Z. et al. *Toxicology Letters* 2006, *163*, 109–20 Lee, I.-C. et al. *International Journal of Nanomedicine* 2016, *11*, 2883–900
Iron oxide	Histopathological lesions in liver and spleen in mice.	Feng, Q. et al. *Scientific Reports* 2018, *8*, article number 2082
TiO_2_	Toxic to liver and brain of mice. Adverse effect on mouse embryos.	Jia, X. et al. *Nanoscale Research Letters* 2017, *12*, article number 478
ZnO	Inflammation and damages to different tissues dependent on the mode of administration in mice or rats.	Gao, L. et al. *Journal of Applied Toxicology* 2013, *33*,1079–88 Wang, D. et al. *International Journal of Occupational and Environmental Health* 2017, *23*, 11–19

## Conclusions and Outlooks

In this review, we have attempted to catalogue the major advances in the preparation, characterization and use of various nanoparticle systems for controlling *S. aureus* growth or replication. So far, the majority of the published studies have involved only *in vitro* testing, and thus, little if anything is known about *in vivo* effects. Several of these, such as the nanosilver materials, have been approved by the U.S. Food and Drug Administration for commercial application. Others are in various stages of clinical trials. Although many nanotherapeutics are potentially exploitable for clinical application, there remain some notable barriers that impede their progression to the market. The major hurdle relates to potential toxicity or other unwanted harmful effects not observable in preclinical trials. These include the buildup of non-degradable particles in nerve cells, fatty tissue, brain matter, bone marrow, or other internal organs.

A second area of concern is the long-term effects of administered nanoparticles on the native microbiome, particularly within the intestinal tract. Each individual has a unique (and fluctuating) microbiome that is vital to our health, but the impact of nanoantibiotics (and antibiotics in general) needs much further evaluation in this regard. Of course, each nanoparticle or nanomaterial is likely to be unique and thus has to be evaluated individually. This requires significant resources and access to clinical trials that are typically well beyond the reach of most research laboratories.

A third concern is that of further expansion of microbial antibiotic resistance, not only of *S. aureus* or MRSA but of all bacteria that are exposed to either the antibiotic or to other microbes that have been treated with the antibiotic. The question is whether the nanoparticles can allow an antibiotic to act equally against the drug-sensitive and the drug-insensitive strains. For instance, even though resistance to silver ion was scarce, as reported, the microorganisms are considered unlikely to develop resistance against silver as compared with antibiotics. The silver nanoparticles interact with multiple targets in the microbial cell, such as cell membrane, enzymes, and plasmids, simultaneously providing the bacteria least capacity to gain resistance. The fact that the drug-resistant and drug-susceptible *S. aureus* strains are affected by silver nanoparticles similarly indicates that the drug-resistant proteins that give bacteria the capacity to avoid antibiotics do not affect the efficacy of nanosilver. Cationic nanoparticles that interact with the negatively charged bacterial membrane often induce cell lysis. To develop resistance, the bacteria would likely need to make rather significant alterations to the membrane surface, and this could make the bacterium more susceptible to other antibiotics (or not survive at all).

Regarding the utility of nanoparticles in the clinical setting, the cost also has to be considered, and whether the use of nanoparticles provides a true benefit over the use of the antibiotic alone. It is a well-known tenet of medicinal chemistry and drug discovery research that despite potent anti-MRSA activity *in vitro*, many antibiotics cannot provide sufficient therapeutic value through the traditional administration routes, mainly because of limited *in vivo* half-lives, toxicity, or poor biodistribution and pharmacokinetics. Nanoparticles as drug delivery vehicles may offer benefit in this case. However, the issues are still more complicated, and largely unexplored, in the realm of nanomedicines. Premature drug release from antimicrobial-loaded nanoparticles through passive diffusion is a major problem, especially for treating systemic and intracellular infections. Our discussion has described how nanoparticles can target antimicrobial agents to the site of infection, so that higher doses of the drug can be given at the infected site, thereby overcoming resistance. To minimize drug loss during transport, nanoparticle carries may be capable of drug release triggers *via* various stimuli such as pH value, enzyme, and other unique characters of the infection microenvironment. Stimulus-responsive nanoparticles such as self-immolative polymeric nanoparticles capable of controlled triggered burst release of small hydrophobic molecules have been recently described, and it would be of interest to design novel anti-MRSA nanoparticles capable of such on-demand release. There is considerable opportunity for development in this area.

In addition, nanoparticles that have the potential to release an antibiotic to intracellular pathogens (such as MRSA) may overcome persistent or re-occurring infections that often lead to bacterial drug resistance. The development of methods to overcome this permeability-mediated resistance is an important therapeutic goal, and nanoparticles that can deliver antibiotics into bacterial cells have also the ability to overcome this resistance.

Combinatorial drug therapy is expected to have higher potency, as multiple drugs can achieve synergistic effects and overwhelm microbial defense mechanisms. One possible approach is to incorporate more than one antimicrobial drug into a single nanoparticle and then concurrently deliver the drugs to the same microbes. A combination of the different strategies such as targeted metallic nanoparticles can also be the solution to efficiently combat MRSA infection with almost no side effects. Although targeted drug delivery has been studied for several infectious diseases, there is a need for the discovery of new specific antigens or particular chemical components of the MRSA cell in order to specifically target that bacteria. Analytical methods for purifying and characterizing nanoparticle materials may be necessary. In particular, often, poorly defined and insufficiently characterized nanoparticles are used for testing, and thus, the correlation of anti-MRSA activity with basic physicochemical properties is made difficult or impossible. There is a great deal of room for investigators to advance nanoantibiotic therapies as a long-term reality for the control and treatment of MRSA infections.

## Author Contributions

Each of the three authors had active roles in researching the literature and compiling the data into the review article. RL is the lead author since he did a significant part of the literature review.

## Conflict of Interest

The authors declare that the research was conducted in the absence of any commercial or financial relationships that could be construed as a potential conflict of interest.
